# Transmigration of Neural Stem Cells across the Blood Brain Barrier Induced by Glioma Cells

**DOI:** 10.1371/journal.pone.0060655

**Published:** 2013-04-05

**Authors:** Mónica Díaz-Coránguez, José Segovia, Adolfo López-Ornelas, Henry Puerta-Guardo, Juan Ludert, Bibiana Chávez, Noemi Meraz-Cruz, Lorenza González-Mariscal

**Affiliations:** 1 Department of Physiology, Biophysics and Neuroscience, Cinvestav, Mexico City, Mexico; 2 Department of Pharmacology, Cinvestav, Mexico City, Mexico; 3 Department of Infectomics and Molecular Pathogenesis, Cinvestav, Mexico City, Mexico; 4 Faculty of Medicine, UNAM, Mexico City, Mexico; 5 National Institute of Genomic Medicine, Mexico City, Mexico; University of Pécs Medical School, Hungary

## Abstract

Transit of human neural stem cells, ReNcell CX, through the blood brain barrier (BBB) was evaluated in an *in vitro* model of BBB and in nude mice. The BBB model was based on rat brain microvascular endothelial cells (RBMECs) cultured on Millicell inserts bathed from the basolateral side with conditioned media (CM) from astrocytes or glioma C6 cells. Glioma C6 CM induced a significant transendothelial migration of ReNcells CX in comparison to astrocyte CM. The presence in glioma C6 CM of high amounts of HGF, VEGF, zonulin and PGE_2_, together with the low abundance of EGF, promoted ReNcells CX transmigration. In contrast cytokines IFN-α, TNF-α, IL-12p70, IL-1β, IL-6, IL-8 and IL-10, as well as metalloproteinases -2 and -9 were present in equal amounts in glioma C6 and astrocyte CMs. ReNcells expressed the tight junction proteins occludin and claudins 1, 3 and 4, and the cell adhesion molecule CRTAM, while RBMECs expressed occludin, claudins 1 and 5 and CRTAM. Competing CRTAM mediated adhesion with soluble CRTAM, inhibited ReNcells CX transmigration, and at the sites of transmigration, the expression of occludin and claudin-5 diminished in RBMECs. In nude mice we found that ReNcells CX injected into systemic circulation passed the BBB and reached intracranial gliomas, which overexpressed HGF, VEGF and zonulin/prehaptoglobin 2.

## Introduction

Neural stem cells (NSCs) constitute a population that continually self-renews and generates the neurons and glia of the brain. NSCs are highly migratory and appear to be attracted to areas of brain pathology. In particular, endogenous neural precursor cells (NPCs) located in the brain subventricular zone have been found to migrate to glial brain tumors [1], where they exert an age dependent antitumorigenic response [2] mediated in part by the release of endovanilloids [3] and bone morphogenetic protein 7 [4]. This ability renders the possibility of using NSC for replacing neurons in degenerative disorders, to repress the proliferation of tumor cells and to deliver therapeutic genes to diseased regions in the brain including minute brain metastasis after main tumor resection [for review see [5]. Thus, NPCs, when systemically injected reach the cerebral parenchyma, induce recovery in animal models of multiple sclerosis [6], and NSCs when implanted into experimental intracranial gliomas *in vivo* in adult rodents, distribute extensively throughout the tumor bed, and when implanted intracranially at distant sites from the tumor, migrate through normal tissue to the tumor cells. What is more, when NSCs are implanted outside of the CNS intravascularly, they are capable of targeting intracranial gliomas [7].

Transendothelial migration of NSCs is regulated by inflammation, reactive astrocytosis and angiogenesis. These processes induce the release of numerous chemokines and growth factors that stimulate the directed migration of NSC towards the site of injury. For example, NPCs express receptors of the chemokines IL-8 and CXL13 and migrate *in vitro* across brain endothelial cells in response to these chemokines [8]. NSC migrate from the contralateral hemisphere towards an infarcted brain area where local astrocytes and endothelium upregulate the expression of stromal cell derived factor 1 (SDF-1)/chemokine CXCL12 [9] and intravenously transplanted NSC migrate to the injured spinal cord in an CXCL12 and hepatocyte growth factor (HGF) dependent manner [10]. In NSC lines, HGH induces the strongest chemotactic response from a variety of multiple tumor-derived growth factors including vascular endothelial growth factor (VEGF), epidermal growth factor (EGF) and transforming growth factor alpha (TGF-α) [11]. VEGF, a growth factor that promotes vasculogenesis, is able to induce long-range attraction of transplanted NSC from distant sites in the brain [12]. Conversely, other factors inhibit NPCs homing. For example, semaphorin 3A/Vascular endothelial growth factor-165 acts as a repellent guidance cue for migrating NPCs [13] and hyaluronic acid, the major ligand of the adhesion molecule CD44, and anti CD44 blocking antibodies prevent adhesion of NPCs to and migration across brain endothelium in inflammatory conditions [6]. In a somewhat similar fashion, hyaluronan accumulates in demyelinated lesions and inhibits the maturation of oligodendrocyte progenitor cells [14].

In order to reach the injuries of the central nervous system, NSCs intravenously injected, need to traverse the brain endothelial cells, which constitute the basis of the blood-brain barrier (BBB). The BBB that limits the entry of blood borne substances into the brain and hence maintains the homeostasis of the CNS, relies on the tight junctions (TJs) present in brain capillaries. The latter are different from those present elsewhere because they display a low rate of fluid phase endocytosis, lack fenestrations and exhibit TJs whose high degree of sealing is regulated by perivascular astrocytes and pericytes [for review see [15]. TJs are constituted by a complex set of integral proteins like claudins, occludin and JAMs, and a group of plaque proteins including cingulin and the ZO proteins 1, 2 and 3 [for review see [16].

Here we studied which factors present in glioma C6 conditioned media induced human NSC to transmigrate across an *in vitro* model of BBB and the impact of these factors on the sealing of TJs in the BBB. We also analyzed the expression of TJ proteins in NSC and in brain endothelial monolayers, and tested how this expression was affected by transmigration. We found that HGF, VEGF, zonulin and PGE_2_ in the absence of EGF in glioma C6 CM induced transmigration, that VEGF, zonulin and PGE_2_ opened the BBB, that ReNcells CX expressed CRTAM, occludin and claudins 1, 3 and 4 that might facilitate their paracellular migration and that at the sites of transmigration the expression of occludin and claudin-5 diminished in brain endothelial monolayers.

## Results

### Glioma C6 cells or CM stimulate the transmigration of NSCs through an *in vitro* BBB system

Our first aim was to determine if NSCs exhibit tropism to glioma C6 cells. For this purpose we plated ReNcell CX on top of Millicell filters with 8 µ pores and analyzed through a migration assay if the presence of glioma C6 cells conditioned media (CM) on the basal compartment stimulated their migration. Figure S1 reveals a significant increased migration of ReNcell CX induced by glioma C6 CM in comparison to CM derived from a primary culture of rat astrocytes or DMEM supplemented with 10% FBS. As expected, CM from ReNcells CX and DMEM did not induce migration.

**Figure 1:**
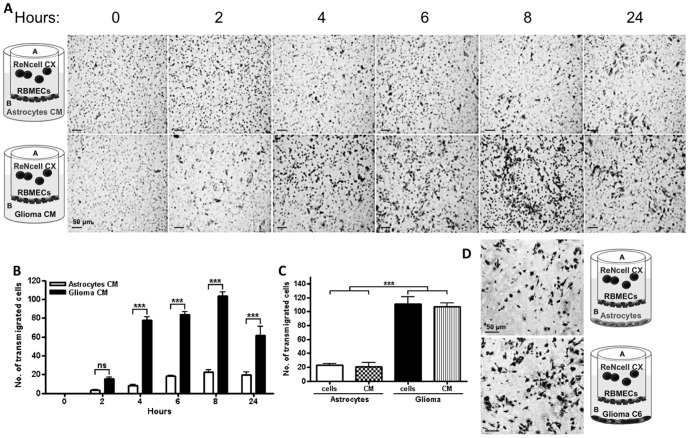
Glioma C6 CM induces transmigration of NSCs across RBMECs. A) Transmigration of ReNcells CX across RBMECs induced by astrocyte or glioma C6 CM. Light microscopy image of toluidine blue stained cells on the basal surface of a filter and scheme illustrating each assay. B) Graphed data. N = 3, F(1,12) = 343.8; *** p<0.001, ns = not significant; as assessed by two-way ANOVA followed by Bonferroni's post hoc test. C) In an 8 h transmigration assay, a similar degree of ReNcells CX transmigration is attained with glioma C6 cells or glioma C6 CM placed in the basal compartment. N = 10, F(3,36) = 60.74; ***P<0.001, ns = not significant; as assessed by one-way ANOVA followed by Bonferroni's post hoc test. D) Representative light microscopy image of toluidine blue stained cells on the basal surface of the filter and scheme illustrating each assay.

Next we tested if glioma C6 CM could induce ReNcells CX transmigration across an *in vitro* BBB model based on the culture of rat brain microvascular endothelial cells (RBMECs). Figures 1A and B show, in a time dependent manner, how the presence of glioma C6 CM on the basal compartment, allowed the transmigration of ReNcells CX across a monolayer of RBMECs. Then we tested in an 8 h transmigration assay if the presence in the basal compartment, of glioma C6 cells, could induce a stronger response than that generated by glioma C6 CM. Figures 1C and D show that the presence of glioma C6 cells or of their CM exerted the same result on ReNcells CX transmigration.

We then analyzed by transmission electron microscopy the transmigration of ReNcells CX. Figure S2 shows the typical elongated spindle shaped morphology of the RBMECs (A and C), how these cells overlapped at the cell borders (B) and grew on top of the pores present in the Millicell filter (C). We also observed ReNcells attached to the apical surface of the RBMECs monolayer (D), bellow the RBMECs monolayers (E) and crossing through the Millicell pore after having traversed the RBMECs monolayer (F).

### Glioma C6 CM decrease the TEER of brain endothelial cells and induce the formation of holes along the cell borders of the endothelial monolayer

**Figure 2:**
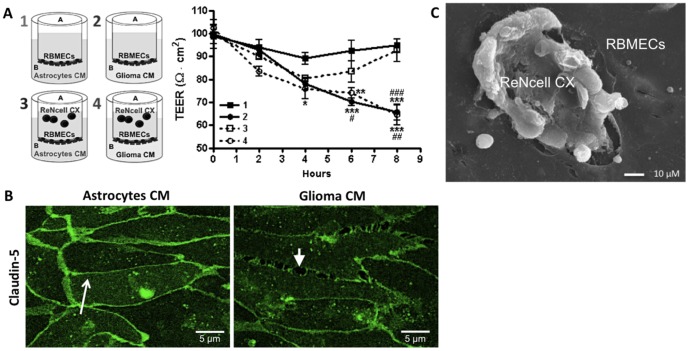
Glioma C6 CM opens the BBB of RBMECs. A) TEER of RBMECs in the conditions illustrated in the scheme. N = 3, F(3,30) = 19.97; *P<0.05, **P<0.01, ***P<0.001, with respect to 1; ^#^P<0.05, ^##^P<0.01, ^###^P<0.001, with respect to 3; as assessed by two-way ANOVA followed by Bonferroni's post hoc test. B) Immunofluorescence localization of claudin-5 and occludin in RBMECs incubated with astrocytes or glioma C6 CM. Arrow, continuous cell border staining; arrowheads, holes appearing at cell borders. C) Scanning electron micrograph of ReNcells CX moving across a hole in the endothelial monolayer.

**Figure 6:**
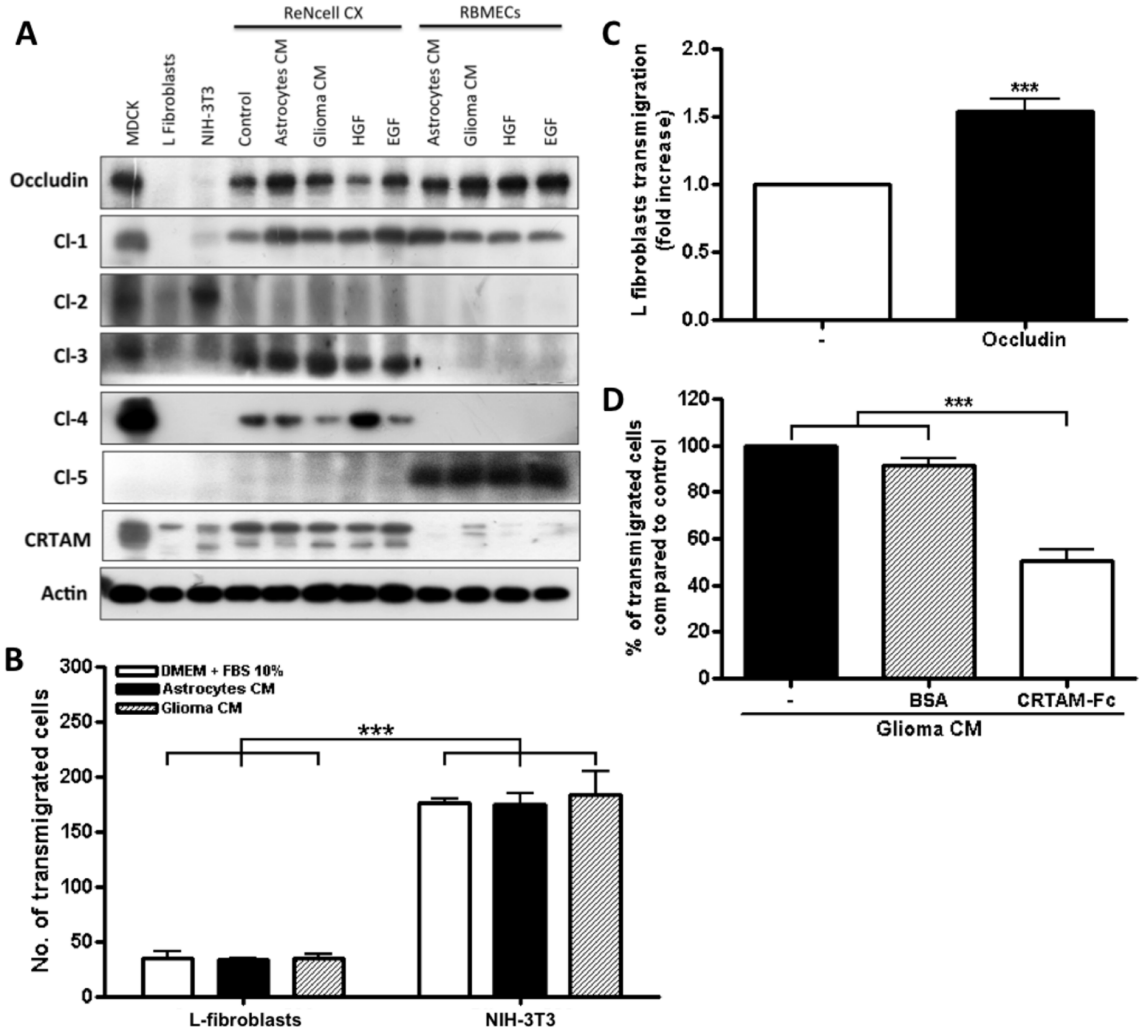
Expression of TJ proteins in transmigrating cells favors their passage across RBMECs. A) Analysis by Western blots of the expression of occludin, claudins 1 to 5 and CRTAM in NIH-3T3 and L-fibroblasts, ReNcells CX and RBMECs. B) The transmigration of NIH-3T3 and L-fibroblasts across RBMECs is independent of the CM present in the basal compartment, but is significantly higher in NIH-3T3 fibroblasts that express claudin-2 than in L-fibroblast that do not express TJ proteins. N = 3, F(1,6) = 232.22, ***P<0.001; F(2,8) = 0.0895, ns = not significant; as assessed by two-way ANOVA followed by Bonferroni's post hoc test. C) Exogenous expression of occludin in L-fibroblasts enhances their transmigration across RBMECs monolayers. N = 9, t = 6.411, df = 8; ***P<0.001 as assessed by Student's t-test. D) Competing CRTAM mediated adhesion with soluble human CRTAM (CRTAM-Fc) added to the upper compartment of a Millicell insert, reduces transmigration of ReNcells CX. N = 4, F(2,6) = 73.86; ***P<0.001, ns = not significant; as assessed by one-way ANOVA followed by Bonferroni's post hoc test.

Another way by which glioma C6 CM could facilitate the transmigration of NSC would be by inducing a leakier monolayer of RBMECs. To test this point we measured the transendothelial electrical resistance (TEER) of RBMECs under the presence of astrocyte or glioma C6 CM in the basolateral compartment. Figure 2A shows that glioma C6 CM induced a sharp decrease in TEER in comparison to monolayers incubated with astrocyte CM. Addition of ReNcells CX to the apical compartment of RBMECs cultures induced a transient decrease in TEER when the basal compartment contains astrocyte CM, whereas the decrease was not reversible when glioma C6 CM was present. Figure 6A shows that incubation with glioma C6 CM exerted no change in the total cellular content of occludin and claudin-5 detected by Western blot, although by immunofluorescence we observed a decreased expression of claudin-5 at the cell borders (Fig. 2B). Most noticeable however, was the appearance of holes along the cell borders of the endothelia incubated with glioma C6 CM (Fig. 2B), and the observation by scanning electron microscopy of ReNcells CX migrating through them (Fig. 2C).

### HGF stimulates NSC transmigration in an *in vitro* BBB system

Since our results indicated that glioma C6 CM promotes the transmigration of ReNcells CX across the RBMECs monolayers, we next sought to identify the factors present in the glioma C6 CM responsible for this effect.

**Figure 3:**
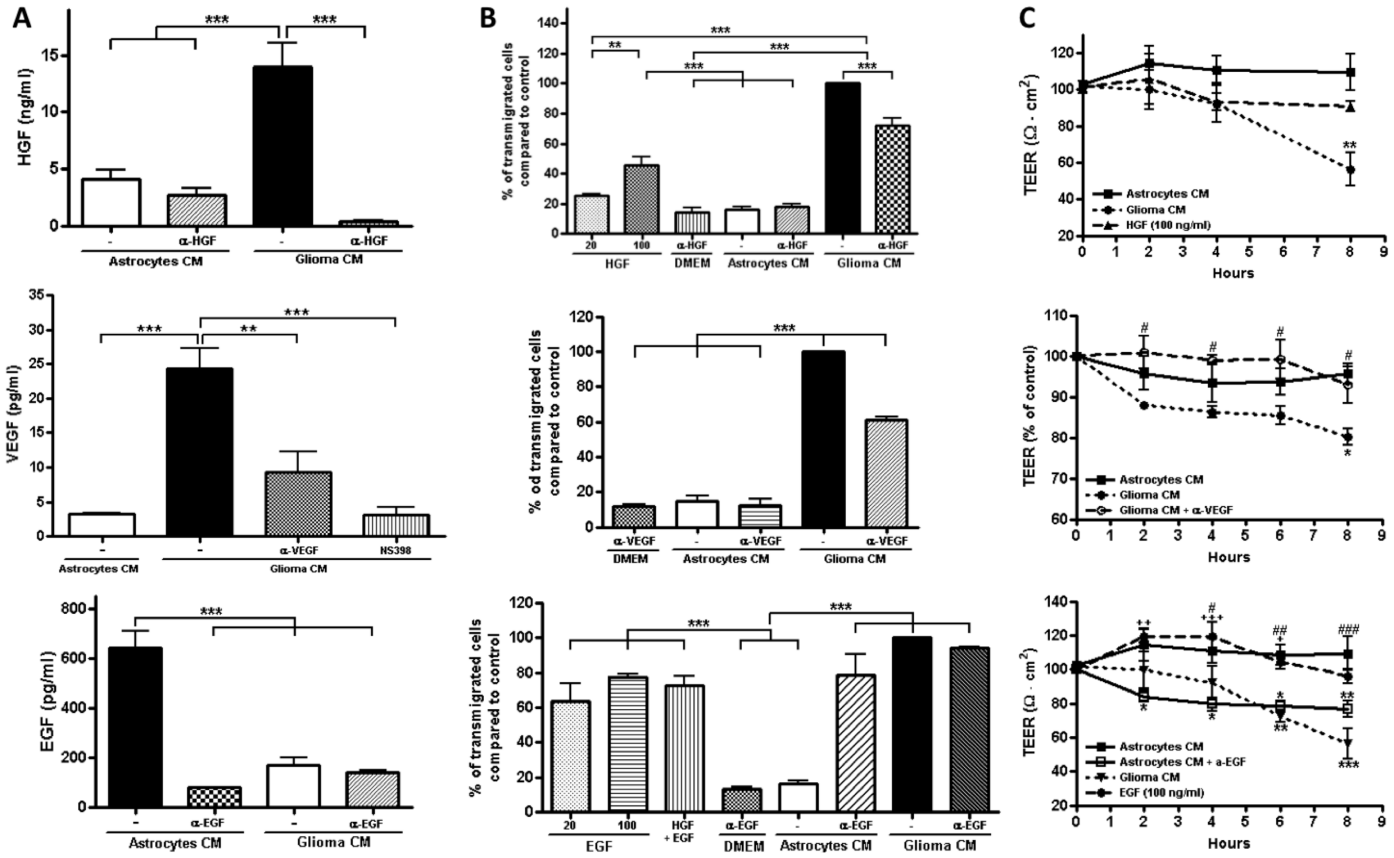
HGF, VEGF and the lack of EGF in glioma C6 CM induce NSCs transmigration across RBMECs. A) The amount of HGF and VEGF determined by ELISA is higher in glioma C6 CM than in astrocyte CM, while the opposite is found for EGF. IP of HGF, VEGF or EGF reduces the amount of the growth factor present in CM. HGF: N = 4, F(3,12) = 26.54; VEGF: N = 3, F(3,8) = 20.98; EGF: N = 3, F(3,8) = 44.52; **P<0.01, ***P<0.001, ns = not significant; as assessed by one-way ANOVA followed by Bonferroni's post hoc test. B) ReNcells CX transmigration assay across RBMECs done by placing in the basal compartment of the Millicell, DMEM with HGF or EGF; or DMEM with neutralizing antibodies against HGF, VEGF or EGF; or astrocyte or glioma C6 CM with or without neutralizing antibodies against HGF, VEGF and EGF. Control corresponds to the transmigration induced by glioma C6 CM without the neutralizing antibodies against HGF (upper panel), VEGF (middle panel) and EGF (lower panel), and is considered as 100% transmigration. HGF: N = 3, F(6,14) = 124.7; VEGF: N = 3, F(4,10) = 275.5; EGF: N = 3, F(7,16) = 30.77; **P<0.01, ***P<0.001, ns = not significant; as assessed by one-way ANOVA followed by Bonferroni's post hoc test. C) RBMECs cultured for 8 h in DMEM with HGF and EGF have a TEER similar to that obtained with astrocyte CM. IP of VEGF reverses the decrease in TEER induced by glioma C6 CM whereas IP of EGF from astrocyte CM decreases TEER in a manner similar to that obtained with glioma C6 CM. HGF: N = 3, F(2,16) = 5.668; **P<0.01 with respect to astrocytes CM; VEGF: N = 3, F(2,20) = 14.64; *P<0.05 with respect to astrocytes CM; #P<0.05 with respect to glioma C6 CM; EGF: N = 3, F(3,30) = 23.07; *P<0.05, **P<0.01, ***P<0.001 with respect to astrocytes CM; ^#^P<0.05, ^##^P<0.01, ^###^P<0.001 with respect to glioma C6 CM; ^+^P<0.05, ^++^P<0.01, ^+++^P<0.001 with respect to astrocytes CM + α-EGF; as assessed by one-way ANOVA followed by Bonferroni's post hoc test.

We started exploring the effect of hepatocyte growth factor (HGF) since this factor has been found to promote the most NSC migration [11]. The amount of HGF was found to be 3.2 times higher in glioma C6 CM than in astrocyte CM (Fig. 3 A). For this purpose we performed a ReNcells CX transmigration assay in which HGF or neutralizing antibodies against it, were added to the basal compartment of the Millicell. Figures S3 A and B show that the addition of 100 ng/ml of HGF to DMEM present in the basal compartment, increased ReNcells CX transmigration in comparison to the condition where astrocyte CM was present in the basal compartment. However, the transmigration induced by HGF was lower than that obtained with glioma C6 CM. To further demonstrate the importance of this factor on glioma-induced transmigration, we added to glioma C6 CM, antibodies with the ability to neutralize the bioactivity of HGF and observed a decreased transmigration of ReNcells CX.

Next, we tested if HGF could alter the TEER of RBMECs cultures. Figure 3C shows that HGF exerted no effect on TEER and in accordance, generated no change in occludin or claudin-5 content (Fig. 6A). These results thus indicate that although HGF exerts a chemotactic effect on ReNcells CX, it does not induce the opening of TJs of RBMECs.

### VEGF stimulates NSC transmigration in an *in vitro* BBB system

Since HGF and vascular endothelial growth factor (VEGF) are the primary chemotactic growth factors produced by gliomas [11], we next analyzed the effect of VEGF on ReNcell CX transmigration. Figure 3 shows that glioma C6 CM had a 7.6 fold higher concentration of VEGF than astrocyte CM and that the amount of VEGF present in glioma C6 CM diminished utilizing a VEGF specific neutralizing antibody. Figure S3 B and figure 3B show a decreased transmigration of ReNcells CX when the anti VEGF antibody was added to glioma C6 CM, confirming the chemo-attractant role of VEGF. The TEER experiment demonstrated that neutralization of VEGF with a specific antibody ablated the decrease TEER generated by glioma C6 CM. Hence, our results indicate that VEGF exerts a chemotactic effect on ReNcells CX and induces the opening of TJs of RBMECs.

### The low amount of EGF present in glioma C6 CM, stimulates NSC transmigration in an *in vitro* BBB system

Epidermal growth factor (EGF) is capable of stimulating NSC migration, although it has been reported that it is not produced in any significant quantity by gliomas [11]. Therefore, in order to explore the effect of EGF in our model we started by determining through an ELISA essay the amount of EGF present in astrocytes and glioma C6 CM. Figure 3A confirmed that astrocyte CM contained a 3.8 fold higher amount of EGF than glioma C6 CM and that a neutralizing antibody against EGF could be employed to deplete the amount of EGF in astrocyte CM. Figure S3 C and figure 3B show that the addition of 20 and 100 ng/ml of EGF to DMEM present in the basal compartment, increased ReNcells CX transmigration in comparison to the condition where astrocyte CM was present in the basal compartment, while the addition of both EGF and HGF did not increase the amount of migrating cells above the level obtained with EGF alone. However, the transmigration induced by EGF was lower than that obtained with glioma C6 CM. To further demonstrate the importance of EGF on transmigration, we added the EGF neutralizing antibody to glioma C6 CM and observed, as expected, no effect since a very low amount of EGF was present in this CM.

When the neutralizing antibody was added to astrocyte derived CM an increased transmigration was observed. In addition, figure 3C shows that EGF induced a transient increase in TEER but without changes in claudin-5 expression, the main component of brain endothelial tight junctions (Fig. 6A). In accordance, treatment of astrocyte CM with EGF neutralizing antibodies decreased the TEER (Fig. 3C). Taken together, these results suggest that the absence of EGF in glioma C6 CM favors a leaky state of the BBB that helps the transmigration or ReNcells CX.

### The presence of PGE_2_ and the lack of EGF in glioma C6 CM promote a leakier BBB

**Figure 4:**
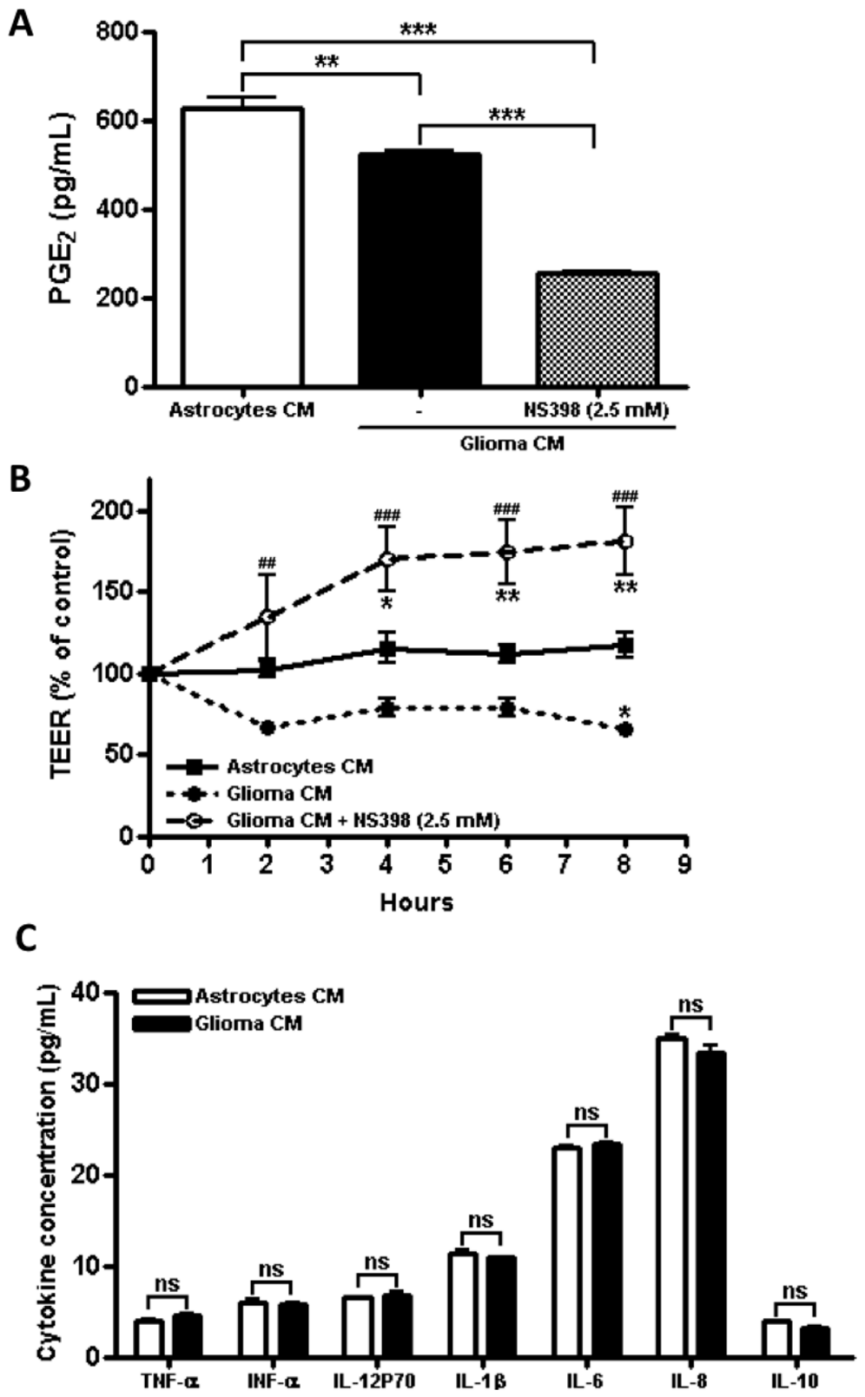
The presence of PGE_2_ and the lack of EGF in glioma C6 CM promote a leakier BBB. A) Quantitation of PGE_2_ by ELISA in astrocyte CM and in glioma C6 CM derived from cells treated or not with COX-2 inhibitor NS398. N = 3, F(2,6) = 161.9; **P<0.01, ***P<0.001; as assessed by one-way ANOVA followed by Bonferroni's post hoc test. B) TEER of RBMECs cultures incubated in the basal compartment with astrocytes or glioma C6 CM derived from control or NS398 treated cells. N = 3, F(2,20) = 53.56; *P<0.05, **P<0.01 with respect to astrocytes CM; ^##^P<0.01, ^###^P<0.001 with respect to glioma C6 CM; as assessed by two-way ANOVA followed by Bonferroni's post hoc test. C) Quantitative analysis of cytokines IFN-α, TNF-α, IL-12p70, IL-1β, IL-6, IL-8 and IL-10 present in astrocyte and glioma C6 CM. N = 3, df = 2, as assessed by Student's t-test.

Prostaglandin E_2_ (PGE_2_) is known to decrease the TEER of epithelia and to inhibit the increase of TEER induced by EGF [17]. Therefore we next tested the amount of PGE_2_ in astrocyte and glioma C6 CM. The quantification of PGE_2_ by an ELISA assay showed a small but significant increase of PGE_2_ in astrocyte CM in comparison to glioma C6 CM (Fig. 4A). However, under the absence of EGF in glioma C6 CM, the presence of PGE_2_ could exert a deleterious effect on the TEER. To test this point, we administered the COX-2 inhibitor NS398 to glioma C6 cells and observed a decreased amount of PGE_2_ in the CM (Fig. 4A) and a pronounced increase in TEER of the endothelial monolayers even above the values obtained with astrocyte CM (Fig. 4B). COX-2 derived PGE_2_ induces VEGF formation [18], therefore treatment of glioma C6 cells with NS398 also inhibited the amount of VEGF present in the corresponding CM (Fig. 3A). Hence the increase in TEER of the endothelial monolayer might be due to the decreased amount of both PGE_2_ and VEGF present in the CM derived from glioma C6 cells treated with the COX-2 inhibitor.

Next we analyzed the amount of cytokines present in astrocyte and glioma C6 CM. The quantitative analysis revealed that IFN-α, TNF-α, IL-12p70, IL-1β, IL-6, IL-8 and IL-10 were present in equal amounts in both CM (Fig. 4C). These results hence suggest that none of these cytokines are responsible for glioma C6 CM induced transmigration of ReNcells CX across the RBMECs monolayers.

### The presence of zonulin/prehaptoglobin-2 in glioma C6 CM promotes BBB opening and ReNcells CX transmigration

**Figure 5:**
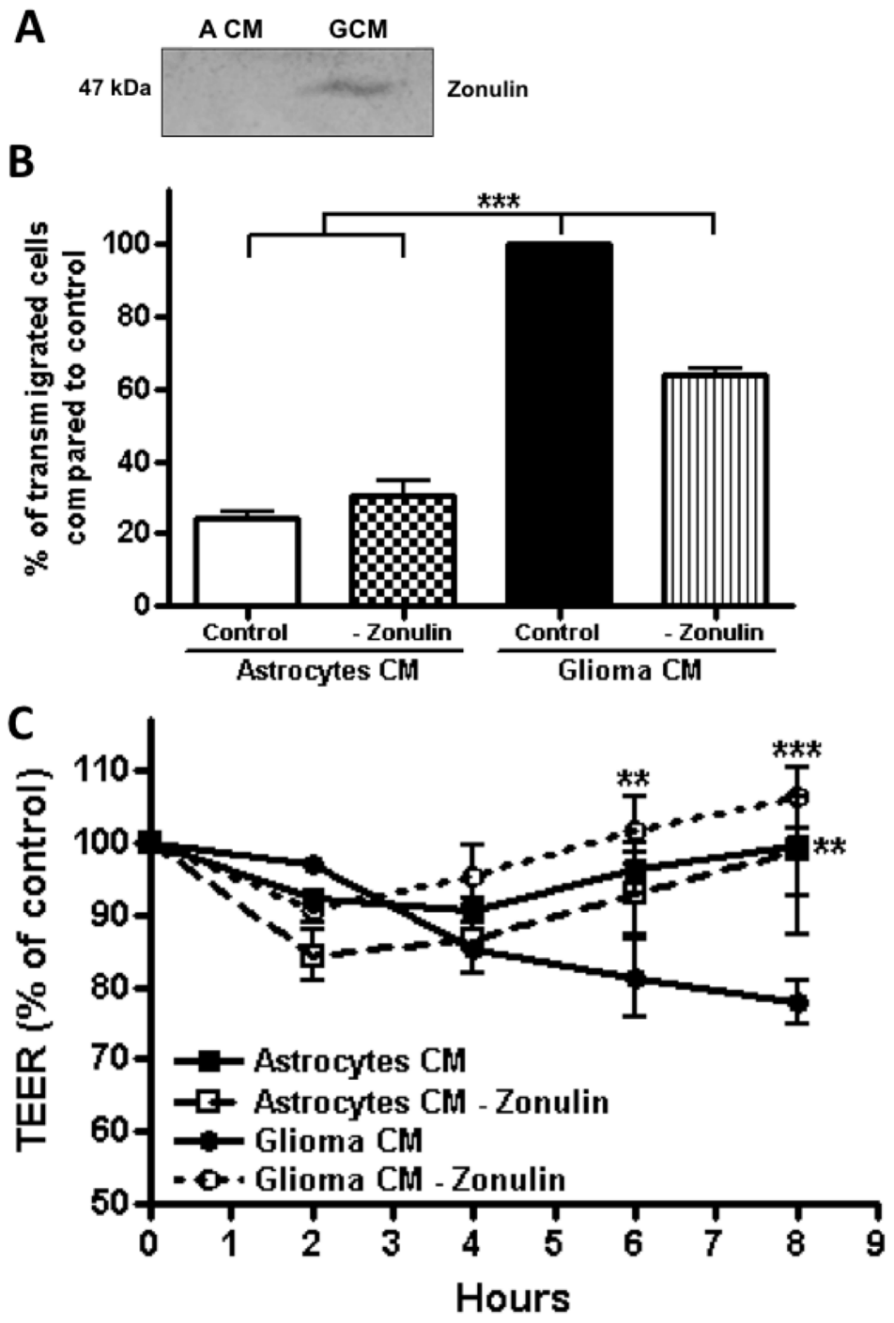
Zonulin present in glioma C6 CM, opens the BBB and favors ReNcells CX transmigration. A) Zonulin is detected by Western blot in glioma C6 CM and not in astrocyte CM. B) ReNcells CX transmigration assay across RBMECs done by placing in the basal compartment of the Millicell, astrocyte or glioma C6 CM with or without zonulin. N = 6, F(3,20) = 228.2; *** p<0.001; as assessed by one-way ANOVA followed by Bonferroni's post hoc test. C) Elimination of zonulin from glioma C6 CM reverses the decrease in TEER exerted by glioma C6 CM. N = 3, F(3,30) = 5.981; **P<0.01, ***P<0.001 with respect to glioma C6 CM; as assessed by two-way ANOVA followed by Bonferroni's post hoc test.

Zonulin recently identified as preheptaglobin-2 [19], is the endogenous eukaryotic analogue of zonula occludens toxin (Zot) from *Vibrio cholera*. Zonulin/preheptaglobin-2 opens the paracellular route by binding to a specific surface receptor that triggers the activation of a signaling pathway that involves phospholipase C and protein kinase C activation, actin polymerization, and contraction of the perijunctional action-myosin ring [for review see [20]. The identification of zonulin/preheptaglobin-2 receptor in the human brain [21], as well as zonulin/preheptaglobin-2 overexpression in gliomas, and its correlation to the degradation of the BBB [22], prompted us to analyze its presence in glioma C6 CM. The Western blot in Figure 5A shows the presence of zonulin/preheptaglobin-2 in glioma C6 CM and not in astrocyte CM. Figure S3 D and figure 5B shows how the elimination of zonulin/preheptaglobin-2 from glioma C6 CM, by immunoprecipitation with an specific antibody, diminished in a 36% the transmigration of ReNcell CX across RBMECs monolayers. This effect appeared to be due to opening of the BBB as the elimination of zonulin/preheptaglobin-2 from glioma C6 CM reverted the decrease in TEER exerted by glioma C6 CM (Fig. 5C).

### A similar amount of metalloproteinases -2 and -9 is present in astrocyte and glioma C6 CM

Our next step was to analyze by gelatin zymography if glioma C6 cells produce metalloproteinases (MMPs) that facilitate the movement of the NSCs across the monolayer of RBMECs. Figure S4 reveals that the CM derived from both astrocytes and glioma C6 cells, contained pro-MMP-2 and to a lesser extent pro-MMP-9. Since these MMPs were present in similar amounts in astrocyte and glioma C6 CM, they do not appear to be the cause of the decreased TEER observed in RBMECs incubated with glioma C6 CM. We next analyzed if the addition of NSC to the endothelial monolayer could alter the zymogram pattern of the various CM. In the second lane of figure S4 we first observed that ReNcells CX CM contains pro-MMP-2 but not pro-MMP-9, while the last four lanes show that the CM from the upper and lower compartments of the Millicell with the RBMEC/ReNcells CX co-culture incubated in the presence of astrocyte or glioma C6 CM, contained pro-MMP-2 and pro-MMP-9. Thus suggesting that the decrease in TEER of the RBMEC/ReNcells CX co-culture obtained after incubation with glioma C6 CM is not due to MMP-2 and MMP-9.

### The exogenous expression of occludin in L-fibroblasts enhances their transmigration across RBMECs monolayers

Our next goal was to test if the presence of TJ proteins in a transmigrating cell could facilitate the cross through brain endothelial cells. For this purpose we worked with L-and NIH-3T3 fibroblasts since the latter contain claudin-2 (Abcam data sheet for rabbit anti claudin-2 antibody AB53032) while the former lack all type of TJ integral proteins [23] (Fig. 6A). Figure S5 A and figure 6B show that NIH-3T3 fibroblasts transmigrated across RBMECs in significantly higher amounts than L-fibroblasts in a manner independent of the CM present in the basal compartment. Since these results suggest that the expression of integral TJ proteins in the transmigrating cell enhances the cross through brain endothelial cells, we next analyzed by Western blot if ReNcells CX express integral TJ proteins. Figure 6A reveals that ReNcells CX expressed occludin and claudins 1, 3 and 4 in all the conditions tested, whereas RBMECs had occludin and claudins 1 and 5. The presence of these TJ proteins might enable NSCs to establish homotypic and heterotypic cell-cell interactions with homologous proteins in RBMECs that could allow the migrating cells to cross the BBB. As proof of principle we next transfected occludin into L-fibroblasts and tested their transmigration. Figure S5 B and figure 6C show a higher transmigration of L-fibroblast expressing occludin in comparison to wild type L-fibroblasts, hence suggesting that the presence of occludin and claudins 1, 3 and 4 in ReNcells CX facilitates their transmigration.

### The expression of occludin and claudin-5 in RBMEC monolayers diminishes around the transmigrating ReNcells

Next we analyzed by immunofluorescence the expression of occludin and claudin-5 in a monolayer of RBMECs during a ReNcell CX transmigration assay. Figures S6 A and B show that the presence of glioma C6 CM in the basal chamber of the Millicell induced the disappearance of occludin and claudin-5 around the transmigrating ReNcells CX stained in red with the cell tracker CMTMR. However by Western blot we were not able to detect any change in the amount of these proteins (data not shown). These results hence suggest that upon basal exposure to glioma C6 CM, ReNcells CX experimented a localized disappearance of TJ proteins at the points of contact between the endothelia and the migrating cells that was not accompanied by a general degradation of these proteins.

### CRTAM mediated adhesion facilitates the transmigration of NSC

Recently, we demonstrated that a novel member of the JAM protein family, named CRTAM is involved in cell-cell adhesion at the lateral membrane of epithelial cells [24]. Therefore, here we tested if CRTAM is expressed by NSCs and participates in their transmigration through RBMECs. Our results in figure 6A, show that CRTAM was present in NIH-3T3 fibroblasts and ReNcells CX and was barely detectable in L-fibroblasts and RBMECs. However, incubation of RBMECs with glioma C6 CM induced its expression albeit at a low level (Fig. 6A). To determine if CRTAM has a role in NSC transmigration, we competed CRTAM mediated cell-cell adhesion by adding soluble human CRTAM (CRTAM-Fc) to the upper compartment of a Millicell insert with ReNcells CX. Figure 6D and figure S7 reveal that soluble CRTAM inhibited the migration of ReNcells CX induced by glioma C6 CM across RBMECs. These results suggest that CRTAM mediated adhesion was important for the transmigration of NSC through brain endothelial cells.

### ReNcells CX injected into systemic circulation target intracerebral gliomas

Our next aim was to test if ReNcells CX injected into systemic circulation were able to reach intracerebral gliomas and to explore under this condition the expression of HGF, VEGF, zonulin/prehaptoglobin-2 and claudin-5. Figure S8 shows that 1 week after injection in the tail vein of mice, ReNcells CX (red) distributed throughout the intracerebral tumor mass formed by glioma C6 cells (green), while no ReNcells CX were found at the contralateral brain hemisphere where glioma C6 cells were not injected, nor in the brain of mice that only received the vehicle, or were non- operated (data not shown). In the tumor area, a strong signal of HGF and slight staining of VEGF was detected, while zonulin/prehaptoglobin-2 and claudin-5 strongly marked the cell borders of surrounding vessels (arrows). In contrast, no HGF was observed in the vehicle section and only a barely above background staining was observed in the contralateral sections; no zonulin was present in the contralateral sections and only a scarce signal was present in the vehicle injected section. With regards to claudin-5, a low intensity spotted signal was observed in the contralateral sections, while in the vehicle section, the cell borders of vessels surrounding the lesion are stained, albeit with low intensity.

The results from the *in vivo* experiment thus demonstrate the capacity of ReNcells CX to pass the BBB and reach intracranial gliomas and confirm the overexpression of HGF, VEGF and zonulin/prehaptoglobin-2 in gliomas.

## Discussion

Malignant gliomas are lethal brain tumors, despite the use of surgery, radiation and chemotherapy. Based on the observation that tumor-derived substances specifically attract stem cells, a novel potential treatment has been developed employing NSC as vectors for the delivery of gene therapy to malignant gliomas [for reviews see [25], [26]. The migration of intravenously injected NSC to intracranial gliomas [7], [27], [28] indicates that these cells have the capacity to cross the BBB. This aspect is particularly relevant since malignant gliomas present in areas of leaky BBB are accompanied by sparse groups of glioma cells deeply infiltrated within the brain parenchyma with an intact BBB. Our aim in this study has been to analyze which factors present in glioma C6 CM induce NSC to transmigrate across an *in vitro* model of BBB and the impact of these factors on the sealing of TJs in the BBB (figure S9).

We have employed an *in vitro* BBB system that consists of a monolayer of RBMECs grown on top of a Millicell chamber with 8 µm diameter pores, in co-culture with rat astrocytes in the basal chamber. Our results indicated that while substitution of astrocytes for astrocyte CM in the basal compartment maintained the TEER, the presence of glioma C6 CM significantly reduced TEER. The transmigration assay demonstrated that ReNcells CX, the human NSC used here, are able to significantly transmigrate across the monolayers of RBMECs when glioma C6 CM is present in the basal chamber.

ELISA and Western blot analysis revealed that VEGF, HGF and zonulin were present in higher amounts in glioma C6 CM than in astrocyte CM, whereas EGF and PGE_2_ were more abundant in astrocyte CM, in agreement with previous observations showing the expression of Zonulin in human gliomas [22], the lack of EGF and the significant production of HGF and VEGF in human glioma cell lines U251 and U87 [11]. Our results showed that VEGF, HGF and zonulin induced transmigration of ReNcells CX through RBMECs monolayers. This can be easily explained for VEGF as it produces a significant chemotactic response in NSC [11] and observed that the neutralization of this factor with an specific antibody in glioma C6 CM induced the recovery of the TEER of the *in vitro* BBB model, thus confirming that VEGF both chemo-attracts and opens TJs [29]. ZOT, the prokaryotic homologue of zonulin, has previously been found to increase the permeability of bovine brain endothelial cells [30], but to our knowledge this is the first time that zonulin has been shown to be involved in the transmigration of cells through brain endothelia. HGF had previously been found to be the most efficient chemo-attractant for NSC produced by glioma cell lines [11]. However, here we found that although HGF induced transmigration of ReNcells CX, it did not decrease TEER upon addition to DMEM. These results indicate that HGF only contributes to transmigration by exerting its chemo-attractant activity.

There is a general agreement that EGF functioning is increased in GBM (glioblastoma multiform), but it is not yet clear whether this is due to over-activity of the receptor (EGFR), or to increased levels of the ligand. The most common gain-of-function mutation in GBM is the amplification and over-expression of the EGFR and this is the major contributor to the invasive phenotype [31]. *EGFR* amplification is found in approximately 50% of GBM and is associated with intragenic rearrangements and/or deletions [32] with the expression of several mutant EGFRs [33]. The receptor EGFR variant III (EGFRvIII) is the most common mutant and is found in ∼40% of GBM in which the EGFR is increased, thus this variant is present in ∼20% of all GBM. This variant increases the invasiveness of tumor, however, it does not bind to its ligand, since it is constitutively activated [34], [35].

EGF levels in serum are elevated in patients with GBM [36] and are also high in primary cultures of monocytes of patients with GBM [37]. Although mutations in the *EGF* gene found in GBM augment its transcription [38], there is a great variation in the levels of EGF released from the tumors which hampers reaching a conclusion as to whether EGF production is decreased or increased in GBM [39].

In the present paper, we determined that EGF induced transmigration of ReNcells CX and increased TEER upon addition to DMEM present in the basal compartment of a Millicell. The answer to this paradox, might be that EGF is capable of inducing chemo-attraction of NSC [11] and that in this condition, in which the BBB is sealed, ReNcells CX instead of crossing through the paracellular pathway are doing it by the transcellular route in a manner similar to that previously reported for neutrophils across the BBB [40]. The increased transmigration of ReNcells CX upon neutralization of EGF present in astrocyte CM, might be due to the opening of the BBB that occurs when PGE_2_ is present in the absence of EGF [17]. This is further reinforced by the observation showing that glioma C6 cells treated with the COX-2 inhibitor, NS398, generate a CM low in PGE_2_ and VEGF that increases TEER to values above those obtained with astrocyte CM.

The depletion by neutralization or immunoprecipitation of VEGF, HGF or zonulin in glioma C6 CM was not sufficient to eliminate ReNcells CX transmigration, thus indicating that no one single factor is responsible for this process and that instead the concomitant participation of all of them contributes to NSC transmigration.

MMPs -2 and -9 do not appear to play a significant role on ReNcell CX transmigration through RBMECs, as they were present in similar amounts in astrocytes and glioma C6 CM.

The mechanism of transmigration across an endothelium includes diapedesis, a process where the migrating cell must move between two or three adjacent endothelial cells. In the case of leukocytes, diapedesis is mediated and facilitated by the expression in both the endothelial and migrating cells of adhesion molecules such as the platelet-endothelial cell adhesion molecule 1 (PECAM-1), CD99 and several adherens junction and TJ molecules including JAMs A, B and C and other proteins related to the JAM family like ESAM, and the nectin-related protein PVR [for review see [41]. In the case of germ cell migration across the paracellular pathway of the seminiferous epithelium during spermatogenesis, it has been observed that several proteins like JAMs, CAR and nectins participate by conferring transient adhesion between Sertoli cells and the migrating germ cells through homophilic and heterophilic interactions [for review see [42]. This background, together with our results showing that the expression of claudins and occludin facilitated the transendothelial migration of fibroblasts, prompted us to suggest that the presence of CRTAM, occludin and claudins in ReNcells CX, might facilitate their transmigration across RBMECs monolayers due to the establishment of homophilic and heterophilic interactions between RBMECs and the migrating ReNcells CX. In particular, the observation that in RBMECs the presence of glioma C6 CM induced the expression of CRTAM, prompted us to explore in more detail the role of this adhesion molecule in ReNcells CX transmigration. The finding that competing CRTAM mediated adhesion with soluble CRTAM (CRTAM-Fc), reduced transmigration of ReNcells CX, is important, as it indicates for the first time that CRTAM mediated adhesion facilitates transendothelial migration.

Glioma C6 CM did not alter the expression of occludin and claudins 1 and 3 in ReNcells CX, but decreased the amount of claudin-4. In RBMECs, glioma C6 CM did not affect the expression of occludin and claudins 1 and 5, but induced the expression of CRTAM. It is thus noteworthy that glioma C6 CM induced in RBMECs, at the sites of ReNcells CX transmigration, a reduced expression of occludin and claudin-5, similar to that observed for claudin-5 in these cells upon melanoma cells transmigration [43] and for occludin in the brain endothelial cell line GP8/3.9 upon monocyte transmigration [44]. This diminished expression of TJ proteins at transmigration sites might respond to a rapid turnover of TJ proteins at these sites in a manner somewhat similar to that observed in the blood-testis barrier of Sertoli cells upon the paracellular migration of spermatogonia [45].

The attraction exerted by glioma C6 CM appears to be specific for NSC, as we observed no transmigration induction in fibroblasts, in agreement with previous observations showing a lack of tropism of fibroblast towards gliomas [7], [46].

In the *in vivo* experiments we demonstrated the capacity of ReNcells CX injected into systemic circulation, to migrate to intracerebral glioma C6 and confirmed our *in vitro* findings, by showing an increased expression of HGF, VEGF and zonulin in the intracranial tumor. We think that in the non-glioma brain sections, HGF was barely detectable and VEGF remained undetectable since these factors were scarcely produced by astrocytes in comparison to gliomas as here shown in the corresponding CM. Since zonulin opens the TJs and is present in tissue damaged by autoimmune and inflammatory disorders, it is not a surprise to find that it was absent from healthy brain sections and was present, albeit in a low degree, in the brain sections that were injured by the intracranial injection with vehicle only. The strong expression of claudin-5 found in the tumor area was expected since glioma growth is angiogenesis dependent [46], while the striatum region of the brain, where the lesion was performed for the injection of ReNcells CX or vehicle, is not as highly vascularized as other regions like the cerebellum, therefore explaining the minimal expression of claudin-5 found in vehicle and contralateral sections.

## Materials and Methods

### Ethics statement

The experimental protocols were approved by The Local Committee of Ethics on Animal Experimentation (CICUAL-Cinvestav) and followed the regulations established in the Mexican Official Norm for the Use and Care of Laboratory Animals (Permit number: NOM-062-ZOO-1999). Rats were euthanized by CO_2_ inhalation. Mice were anesthetized with sodium pentobarbital for surgery and injection, and deeply anesthetized and perfused to obtain brain sections. All efforts were made to minimize suffering.

### Cell lines and primary cultures

The immortalized human neural progenitor cell line ReNcell CX, derived from the cortical region of human fetal brain and with the ability to differentiate into neuron and glial cells, was obtained from Merck Millipore (Billerica, M.A., SCC007) and cultured as indicated by the provider. Briefly, cells were grown in ReNcell CX medium (Merck Millipore, SCM005), supplemented with EGF, bFGF (20 ng/ml) (Sigma Aldrich, St. Louis, M.O.), in the presence of 1% penicillin/streptomycin (In Vitro, Mexico D.F., A-01) at 37°C in 5% CO_2_.

Glioma C6 cell line, developed by repetitive administration of methylnitrosourea in adult Furth rats, was obtained from the ATCC (CCL-107) and cultured in DMEM/F12 (Invitrogen, Carlsbad, C.A., 12500-062) supplemented with 10% FBS and antibiotics (penicillin 100 U/ml, streptomycin 100 mg/ml).

Mouse L-fibroblasts and NIH-3T3 were obtained from the ATCC (CCL-1; CRL-1658) and cultured in DMEM (Invitrogen, 31600-026) supplemented with 10% FBS and antibiotics (penicillin 100 U/ml, streptomycin 100 mg/ml). 0.08 U/ml of insulin (Eli Lilly and Company, Indianapolis, I.N., 074M90) was additionally supplemented to the L-fibroblast media.

Primary culture of rat astrocytes from the cerebral cortex of newborn rats was done as previously described [47]. Briefly, the meninges of 2-day old Wistar rat brains were removed, the cerebral cortices placed into 4 ml of DMEM and cut into small pieces and digested with 1.25 mg/ml trypsin (Invitrogen, 15050-065) for 10 min. Then the medium was changed for fresh one and the cells were gently resuspended 15 times with a 5 ml micropipette tip in a 15 ml Falcon tube. After 3 min the supernatant was gently forced through a 40 µm nylon cell strainer (BD Biosciences, Franklin Lakes, N.J., 352340). The procedure was repeated until 24 ml of the supernatant had passed through the sieve. The filtered cell suspension was then plated into 12 well plates coated with 10 µg/ml poly-L-lysin hidrobromide (Sigma Aldrich, P6282) in DMEM with 10% FBS and antibiotics (penicillin 100 U/ml, streptomycin 100 mg/ml, 1 µg/ml gentamycin, 1 µg/ml kanamycin and 1 µg/ml fungizone). The medium was changed every three days.

Primary rat brain microvascular endothelial cells (RBMECs) were isolated from 2 week old Wistar rats as previously described [48]. Briefly, the meninges were removed and the cerebral cortices cut into small pieces and digested with 1 mg/ml collagenase type 2 (Sigma Aldrich, C6885) at 37°C for 75 min. Myelin was then separated by centrifugation for 20 min at 1000 g in 20% BSA, and a second digestion was performed with 1 mg/ml collagenase/dispase (Roche Diagnostics, Indianapolis, I.N., 11097113001) at 37°C for 50 min. Fragmented microvessels were collected after a 10 min centrifugation at 1000 g on a Percoll (Sigma Aldrich, P1644) gradient, and plated onto fibronectin/collagen coated dishes. The endothelial cells growing out of the microvessels were cultured in DMEM/F12 containing 20% plasma derived serum (Animal Technologies Inc., Tyler, T.X., 11-090423), 500 ng/ml bFGF (Bioworld, Atlanta, Georgia, 507361), 0.2 M Glutamax (Invitrogen, 35050079), 8 µg/ml heparine (Lab. Pisa S.A. de C.V., Guadalajara, Jal., Mexico, 177M90 SSA IV) and antibiotics [penicillin 100 U/ml, streptomycin 100 mg/ml, 1 µg/ml gentamycin (Schering-Plough S.A. de C.V., Mexico D.F., 63671 SSA IV), 1 µg/ml kanamycin (Bistrol-Myers Squibb de Mexico, S. de R.L. de C.V., Mexico, D.F., 53508 SSA IV) and 1 µg/ml fungizone (Invitrogen, 15290-018)]. During the first two days of the culture, 1 µg/ml of puromycin (Sigma Aldrich, P9620) was added to remove contaminating cells.

### Conditioned media (CM)

CM was obtained from two sources: a) from four day old cultures of ReNcells CX, RBMECs, astrocytes, and glioma C6 cells, and b) from the upper and lower compartments of a Millicell, 8 hours after ReNcells CX were plated onto the RBMEC monolayer in the presence of astrocyte or glioma C6 CM in the basal compartment.

In some transmigration and transendothelial electrical resistance (TEER) experiments the CM from astrocytes and glioma C6 cells were incubated for 1 h with the following neutralizing antibodies previously dissolved in PBS: anti rat HGF (Abcam, Cambridge U.K., Ab10679, concentration 8 µg/ml), anti rat VEGF (Peprotech, Rocky Hill, N.J., 500-P275, concentration 0.1 µg/ml) and anti rat EGF (Peprotech, 500-P277, concentration 0.1 µg/ml). The mouse monoclonal antibody against zonulin (Abcam, Ab118056, dilution 1:250) was employed to immunoprecipitate zonulin from astrocyte and glioma C6 CM. Astrocyte or glioma C6 CM lacking zonulin, containing the growth factor neutralizing antibodies, or having the expression of MMPs inhibited, were placed in the basal chambers of Millicell wells.

ELISA assays were done in CM from astrocytes and glioma C6 cells previously depleted by immunoprecipitation of growth factors, with specific antibodies against EGF (dilution 1:250) or VEGF (dilution 1:250). To obtain a glioma C6 CM with a reduced expression of PGE_2_, glioma C6 cells were treated 24 and 72 hours after plating with 2.5 µM of COX-2 inhibitor NS398 (EMD Biosciences, La Jolla, C.A., 349254), and the CM was collected 4 days after plating.

### Measurement of transendothelial electrical resistance (TEER)

RBECs were grown on inserts with semipermeable filters (8.0 µm pore size, 0.6 cm^2^ Millicell standing cell culture 24 well PCF, Millipore, PI8P01250; or 8.0 µm pore size, 1.12 cm^2^ Millicell hanging culture 12 well PET, Millipore, PIEP15R48). After the cultures reached confluency, the endothelial monolayers were supplemented at both the upper and lower compartments with 12.5 mg/ml 8-(4-Chlorophenylthiol) adenosine 3′,5′-cyclic monophosphate sodium salt (Sigma Aldrich, C3912), 9.75 mg/ml the c-AMP phosphodiesterase-4-specific inhibitor RO-20-1724 (Sigma Aldrich, B8279) and 50 µg/ml hydrocortisone (Sigma Aldrich, HO888) and placed into the wells of the CellZcope*®* instrument (nanoAnalytics, Münster, Germany) or in a multiwell plate containing CM from 4 days of culture of astrocytes or glioma C6 cells. TEER was measured either in the CellZcope or using the voltohmmeter EVOM (Word Precision Instruments, New Haven, C.T.). To analyze the effect on TEER of the transmigration of ReNcell CX, the latter were plated in the apical chamber.

### Migration assays

ReNcell CX cells were left overnight without growth factors, incubated with accutase (Sigma Aldrich, A6964), resuspended in serum free ReNcell media and plated at a density of 1×10^5^ cells/cm^2^ onto Millicell inserts with 8 µm pores in 24 well plates, containing or not a monolayer of RBMECs. In some experiments L-fibroblasts or NIH-3T3 with or without chicken occludin (generously provided by Maria Susana Balda, University College, London, U.K.) transfected with Lipofectamine™ 2000 (Invitrogen, 11668019), were added to the apical surface of RBMECs plated on Millicell inserts.

For the migration assay, the lower chamber contained DMEM, DMEM with 10% FBS with or without hEGF (Sigma Aldrich, E9644) or hHGF (Peprotech, 1039), primary cultured astrocytes, glioma C6 cells, or CM derived from ReNcells CX, astrocytes or glioma C6 cells. In some experiments recombinant human soluble CRTAM (CRTAM-Fc, R&D Systems, Minneapolis, M.N., 1695-CR) was added at a concentration of 20 µg/ml to the apical compartment of Millicell inserts.

The number of migrating cells was evaluated 8 h later by fixing the cells with 3% paraformaldehyde for 20 min, permeabilized with 0.03% saponin and then removing the non-migrated cells in the upper well with a cotton swab. Then the filters were immersed in 1% toluidine blue for 10 min, washed thrice in water, cut from the inserts, and observed in a light microscope (Nikon Eclipse E600, Japan). The number of toluidine blue stained cells present on the basal surface of the Millicell filter was counted using ImageJ on 16 optical fields per filter and in three filters per experimental condition.

### Transmission electron microscopy

Millicell inserts (8 µm pores), with a cultured monolayer of RBMECs and ReNcells CX placed on top and induced to transmigrate after an 8 hour incubation with glioma cells CM on the basal compartment, were fixed with 2.5% glutaraldehyde in 0.1 M sodium cacodylate buffer, pH 7.2 during 1 hour and post-fixed with 1% osmium tetroxide for 1 hour. After dehydratation in graded ethanols and propylene oxide, the filters were detached from the culture dish by cutting the border with a knife, embedded in eponate resin and polymerized at 60 °C during 24 hours. Thin, 60 nm sections were contrasted with uranyl acetate and lead citrate for observation in a Zeiss EM910 electron microscope.

### Scanning electron microscopy

RBMECs monolayers grown in Millicell membranes were fixed for 1 hour with 2.5% glutaraldehyde in 0.1 M sodium cacodylate buffer pH 7.4, containing 0.1 M sucrose. The membrane with the monolayer was then cut with a knife and post-fixed for 1 h with osmium tetroxide in the same buffer. Dehydration was carried out in a graded series of ethanol concentration. The last 100% ethanol solution was replaced with hexamethyldisilazane (HMDS) (Polysciences Inc., Warrington, P.A.). After 10 min HMDS was replaced for fresh HMDS and 10 min later HDMS was removed and the samples were left to air-dry for 30 min. Samples were mounted in specimen metal mounts and gold coated in a ion sputtering devise (JEOL-JFC-1100). Samples were examined in a JEOL scanning electron microscope (JSM-6510-LV).

### Western blot

Western blots were done following standard procedures previously described by us [49] and using rabbit IgG polyclonal antibodies against occludin (Invitrogen, Cat. 71-1500, dilution 1:1000), claudin-1 (Invitrogen, Cat. 51-9000, dilution 1:500), claudin-2 (Invitrogen, Cat. 51-6100, dilution 1:250) and claudin-3 (Invitrogen, Cat. 34-1700, dilution 1:250); and mouse monoclonal antibodies against claudin-4 (Invitrogen, Cat. 32-9400, dilution 1:250), claudin-5 (Invitrogen, Cat. 187364, dilution 1:1000), CRTAM (R&D Systems, Cat. Mab1695, dilution 1:250), zonulin (Abcam, Cat. Ab118056, dilution 1:500) and actin (a generous gift of Dr. Jose Manuel Hernández, Department of Cell Biology, Cinvestav, México, D.F., dilution 1:2000). As secondary antibodies the following peroxidase-conjugates were used: goat Ig anti-mouse IgG (Zymed Laboratories, Grand Island, N.Y., Cat. 62-6420, dilution 1:5000); goat Ig anti-rabbit IgG (Zymed Laboratories, Cat. A9169, dilution 1:5000). Followed by a chemiluminiscence detection system (ECL + PLUS, GE Health-Care, Piscataway, N.J., Cat. RNP2132).

### Cytokine detection

Cytokine quantification in 4 day CM derived from astrocytes and glioma C6 cells was done using a cytometric bead array (BD™ CBA Flex Set, BD Biosciences, US Cat. 558264) that employs particles with discrete fluorescence intensities to detect soluble analytes The Flex Set included seven cytokines with detection limit expressed in pg/mL as follow: human IL-8 (1.2) (Cat. 558277), IFN-α (1.5) (Cat. 560379), IL-1β (2.3) (Cat. 558279), TNF-α (0.7) (Cat. 558273), IL-12p70 (0.6) (Cat. 558283), IL-10 (0.13) (Cat. 558274), IL-6 (1.6) (Cat. 558276). Briefly, culture supernatants were simultaneously immunoprecipitated by using a captured-bead array coated with specific antibodies. Then, a phycoerythrin-conjugated detection reagent was added and finally a flow cytometry assay was carried out. Data acquisition and analyses were done in a BD FACSCalibur™ flow cytometer and BD CellQuest™ Pro Software (BD Biosciences, U.S., 342976). Supplied standards were used to construct standard curves (*R^2^>0.9957*) and mean fluorescence intensity (MIF) results allowed sample analysis and soluble protein quantification by Microsoft*®*Excel.

### Detection of VEGF, HGF, EGF and PGE_2_

Quantification was done in 4 day CM derived from astrocytes and glioma C6 cells using solid phase sandwich ELISA for VEGF (Invitrogen, KHG0111), HGF (Ray Biotech Inc., ELM-HGF-001), EGF (Peprotech, 900-M390) and PGE_2_ (ENZO Life Science, Farmingdale, N.Y., ADI-900-001).

### Zymography

CM were collected and centrifuged at room temperature for 2 min. The supernatant were then concentrated using an Amicon ultra-0.5 centrifugal filter unit with ultracel-30 membrane (Millipore, UFC503096). Samples were next electrophoresed under non-denaturing conditions in 8% polyacrylamide gels containing 1% gelatin. To determine the proteolytic activity, gels were washed twice for 15 min in a solution containing 2.5% Triton X-100 and incubated in a buffer containing 50 mM Trizma Tris base (pH 7.4), 150 mM NaCl and 20 mM CaCl_2_. Gelatinase activity was visualized by staining with 0.25% Coomassie brilliant blue R-250 in 45% methanol and 10% acetic acid and destained in a solution of 20% methanol and 10% acetic acid. The supernatant of cultured U937 pro-myelocytes, was used as a standard of enzymatic activity for MMP-2 and MMP-9 as previously reported [50].

### Immunofluorescence

Immunofluorescence was done on: 1) monolayers of RBMECs cultured on glass coverslips, incubated for 8 h with astrocyte or glioma C6 CM, and 2) transmigration assays. For the latter ReNcells CX in culture were stained with the CellTracker™ Orange CMTMR (Invitrogen, C2927) following the manufacturer's instructions. Then, ReNcells CX were lifted with accutase, resuspended and plated at a density of 1×10^5^ cells/cm^2^ on top of RBMECs monolayers cultured on Millicell inserts with astrocyte or glioma C6 CM present in the basal chamber. After 8 h, the upper chamber of the Millicell was gently washed with PBS to eliminate ReNcells CX that had not adhered to RBMECs monolayers.

Immunofluorescence was done following standard procedures [51] in cultures fixed with 4% paraformaldehyde and permeabilized with 0.25% Triton X-100, employing a rabbit polyclonal antibody against occludin (Invitrogen, Cat. 71-1500, dilution 1:100) and a mouse monoclonal antibody against claudin-5 (Invitrogen, Cat. 187-364, dilution 1:100), and FITC conjugated secondary antibodies from goat against rabbit IgG (Zymed, Cat. 62-6111, dilution 1:300) and against mouse IgG (Zymed, Cat. 62-6511, dilution 1:300). Samples were observed in a Leica SP-2 confocal microscope with Argon and Helium-Neon lasers employing the Leica confocal software.

### *In vivo* migration of ReNcells CX to intracranial gliomas

For *in vivo* migration experiments, we employed nude mice (*nu/nu*), glioma C6 cells pre-infected with a retrovirus containing the Green Fluorescent Protein (GFP), sequence and sorted by flow cytometry as previously described [52] and ReNcell CX stained with the CellTracker™ Orange CMTMR. The day of surgery mice were anesthetized with Ketamine 100 mg/kg and Xylazine 10 mg/kg, and 1×10^6^ glioma C6 tumor cells expressing the GFP, resuspended in 3 µl of DMEM/F12, were stereotaxically injected into the striatum (Bregma AP: + 0.5 mm; ML: 2 mm and DV: 3 mm). Two controls were used, one received only vehicle (3 µl of DMEM/F12; lesion), and the second was non-operated (intact). Seven days later, mice were anesthetized and 1.5×10^6^ ReNcells CX stained with the CellTracker™ orange CMTMR, were resuspended in 200 µl of ReNcell medium without factors and injected in the tail vein. After one week, mice were deeply anesthetized and perfused with saline containing heparin, 1000 U/ml, and fixed with paraformaldehyde 4%. Finally, 30 µm coronal sections were obtained from the brains and observed using laser confocal microscopy (Leica Microsystems).

Immunohistochemistry was done to detect the presence of HGF, VEGF, zonulin and claudin-5. Slices were washed 3 times with PBS 1X and then permeabilized with PBS-Triton (0.25%) for 30 minutes. Subsequently were blocked with PBS-Triton with BSA (1%) for one hour and primary antibodies were incubated for 24 hours. One day later, slices were washed 3 times with PBS 1X and incubated with the secondary antibodies for 24 hours. Lastly, slices were washed 5 times with PBS 1X.

## Conclusions

Considering that NSC might potentially be used as vehicles for targeting therapeutic genes to gliomas, we have characterized in an *in vitro* BBB model the factors controlling NSC transmigration induced by glioma C6 CM. We have found that HGF, VEGF, zonulin and PGE_2_ in the absence of EGF in glioma C6 CM induce transmigration, that VEGF, zonulin and PGE_2_ open the BBB, that ReNcells CX express CRTAM, occludin and claudins 1, 3 and 4 that might facilitate their paracellular migration and that at the sites of transmigration the expression of occludin and claudin-5 diminishes in RBMECs. In nude mice we found that ReNcells CX injected into systemic circulation, pass the BBB and reach intracranial gliomas, which overexpress HGF, VEGF and zonulin/prehaptoglobin-2.

## Supporting Information

Figure S1 **Glioma C6 CM induces the migration of NSCs.** A) A representative light microscopy image of toluidine blue stained cells present on the basal surface of the filter is shown together with a scheme illustrating each assay. B) Graphed data. N = 4, F(4,15) = 40.64; *P<0.05, ***P<0.001; as assessed by one-way ANOVA followed by Bonferroni's post hoc test. The ReNcells CX migration assays were done on Millicell filters with 8 µm pores.
(TIF)

Figure S2 **Transmission electron microscopy of a monolayer of RBMECs with transmigrating NSCs.** A) Monolayer of RBMECs cultured on a Millicell filter with 8 µm pores. Left panel, observe the typical elongated morphology of an endothelial cell. Middle panel, border region between two neighboring endothelial cells; observe how the cells partially overlap. Right panel, view an elongated endothelial cell on top of a pore of the Millicell filter. B) ReNcells CX crossing a monolayer of RBMECs. Upper left panel, observe a ReNcell CX on top of two neighboring RBMECs. Lower left panel; observe two ReNcells CX located bellow the monolayer of RBMECs. Right panel, a ReNcell CX is found crossing through a Millicell pore. *, Millicell filter; arrow, paracellular pathway. EC1, endothelial cell one; EC2, endothelial cell two; P, Millicell pore. Bar  =  1 µm.
(TIF)

Figure S3 **HGF, VEGF, zonulin, and the lack of EGF induce NSCs transmigration across RBMECs cultures.** Representative light microscopy image of toluidine blue stained cells present on the basal surface of the filter and corresponding schemes illustrating each assay after the addition to the basal compartment of HGF or its neutralizing antibody (A), a neutralizing antibody against VEGF (B); EGF or its neutralizing antibody (C), and CM without zonulin due to specific IP (D)
(TIF)

Figure S4 **CM from glioma C6 and astrocytes have similar amounts of pro-MMP-2 and -9.** CM derived from different cell cultures (ReNcells CX, RBMECS, astrocytes and glioma C6 cells) and from the co-cultures shown in the right hand side were electrophoresed in non-denaturing conditions in 8% polyacrylamide gels containing 1% gelatin. Proteolytic activity was induced by incubation in a Ca^2+^ containing buffer and proteolytic bands were visualized by Coomassie blue staining. CM from melanoma U937 cells was employed as standard of MMP-2 and -9 activities. A1CM, CM of assay A present in compartment 1; A2CM, CM of assay A present in compartment 2; B1CM, CM of assay B present in compartment 1; B2CM, CM of assay B present in compartment 2.
(TIF)

Figure S5 **The expression of TJ proteins in fibroblasts enhances their transmigration across RBMECs.** Transmigration assay with toluidine blue stained cells A) NIH-3T3 fibroblasts that express claudin-2, transmigrate across RBMECs in significantly higher amounts than L-fibroblasts, which do not express TJ proteins, independently of the CM present in the basal compartment. B) L-fibroblast that express occludin, transmigrate across RBMECs in significantly higher amounts than L-fibroblasts.
(TIF)

Figure S6 **In RBMEC monolayers incubated with glioma C6 CM in the basal compartment, the expression of occludin and claudin-5 diminishes around the transmigrating ReNcells.** Occludin (A) and claudin-5 (B) were detected with a specific antibodies followed by secondary antibodies coupled to FITC. Transmigrating ReNcells CX were stained in red with the cell tracker CMTMR. Arrows point to areas where occludin and claudin-5 expression is lost.
(TIF)

Figure S7 **CRTAM mediated adhesion is important for the transmigration of NSC.** CRTAM mediated cell-cell adhesion was competed by adding soluble human CRTAM (CRTAM-Fc) to the upper compartment of a Millicell insert with ReNcells CX. Upper left panel, scheme illustrating each assay; lower left panel, representative light microscopy image of toluidine blue stained cells present on the basal surface of the filter.
(TIF)

Figure S8 **ReNcells CX injected into systemic circulation pass the BBB and reach intracerebral gliomas.** Immunofluorescence detection (blue) of HGF, VEGF, zonulin/prehaptoglobin-2 and claudin-5, in brain slices of the stratum region of nude mice. The animals had previously been injected into the striatum with vehicle or glioma C6 cells containing the GFP sequence (green) and a week later received ReNcell CX stained with CMTMR (red) by injection into the tail vein. Brain slices were done one week later. Only at the tumor area, a strong signal of HGF and slight staining of VEGF is observed, while zonulin and claudin-5 strongly mark the cell borders of surrounding vessels (arrows). Zonulin is also present, albeit with very low intensity, in the area of the lesion in brains that had only received the vehicle (arrowhead). Claudin-5 gives a spotted pattern in the contralateral section (arrowhead) and stains, albeit with low intensity, the vessels in the vehicle only section (arrowhead).
(TIF)

Figure S9 **Schematic representation of the factors involved in the transmigration of NSC across the BBB.** HGF, VEGF and zonulin secreted by glioma C6 cells, together with the absence of EGF, induce the transmigration of ReNcells CX across RBMECs. VEGF, zonulin, PGE_2_, and MMP different from -2 and -9, secreted by glioma C6 cells, open the BBB, whereas EGF secreted by astrocytes enhances TJ sealing. ReNcells CX express CRTAM, occludin and claudins 1, 3 and 4 that might facilitate their paracellular migration across RBMECs that have TJs formed by CRTAM, occludin and claudins 1 and 5.
(TIF)
