# Supplementary figures and images for: Transmigration of Neural Stem Cells across the Blood Brain Barrier Induced by Glioma Cells
Source: PLoS One. 2013 Apr 5;8(4):e60655. doi: 10.1371/journal.pone.0060655 (PMC3618035; doi:10.1371/journal.pone.0060655)

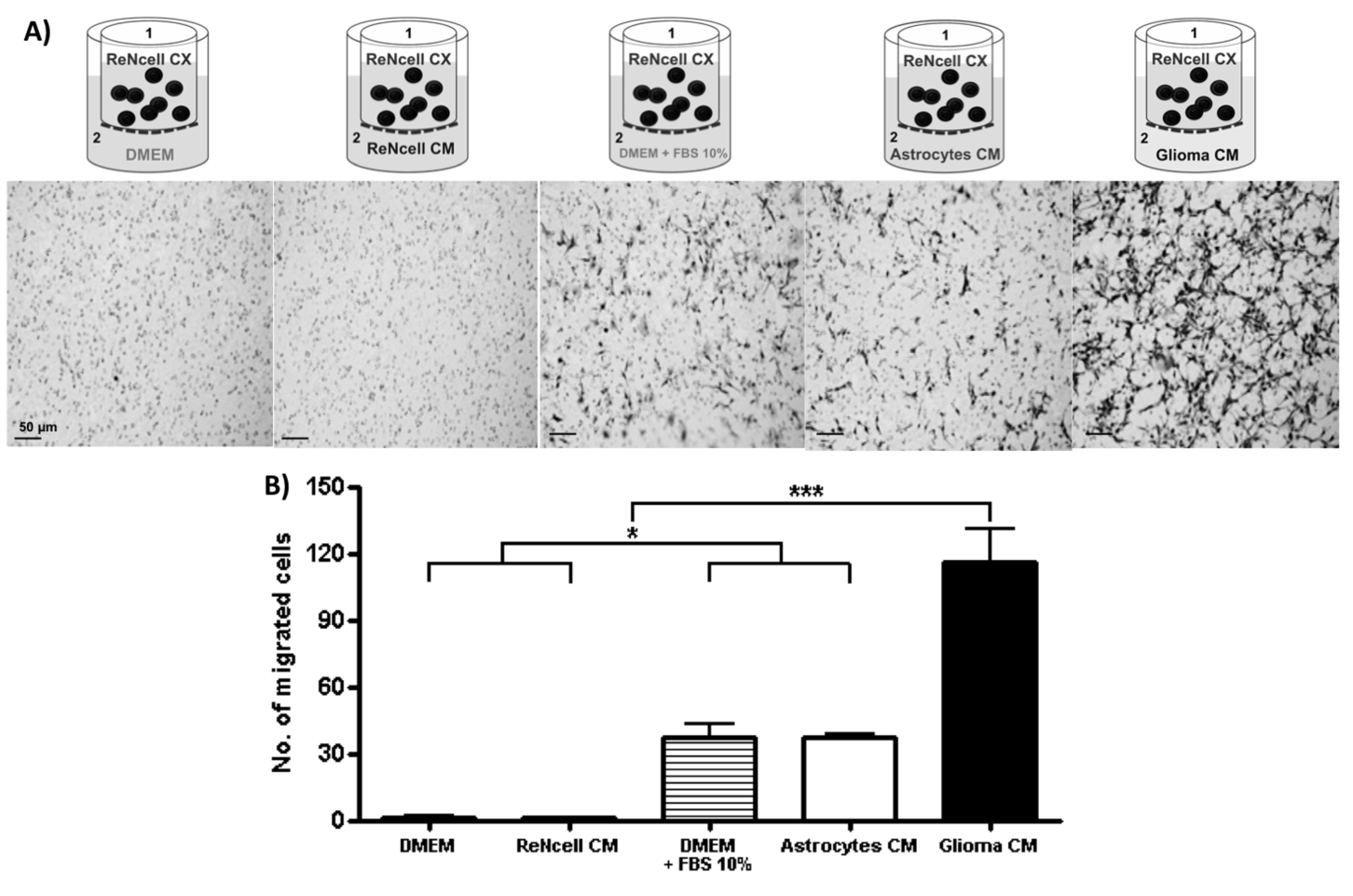

Supplement: Figure S1 — Glioma C6 CM induces the migration of NSCs. A) A representative light microscopy image of toluidine blue stained cells present on the basal surface of the filter is shown together with a scheme illustrating each assay. B) Graphed data. N = 4, F(4,15) = 40.64; *P<0.05, ***P<0.001; as assessed by one-way ANOVA followed by Bonferroni's post hoc test. The ReNcells CX migration assays were done on Millicell filters with 8 µm pores. (TIF) [file pone.0060655.s001.tif]

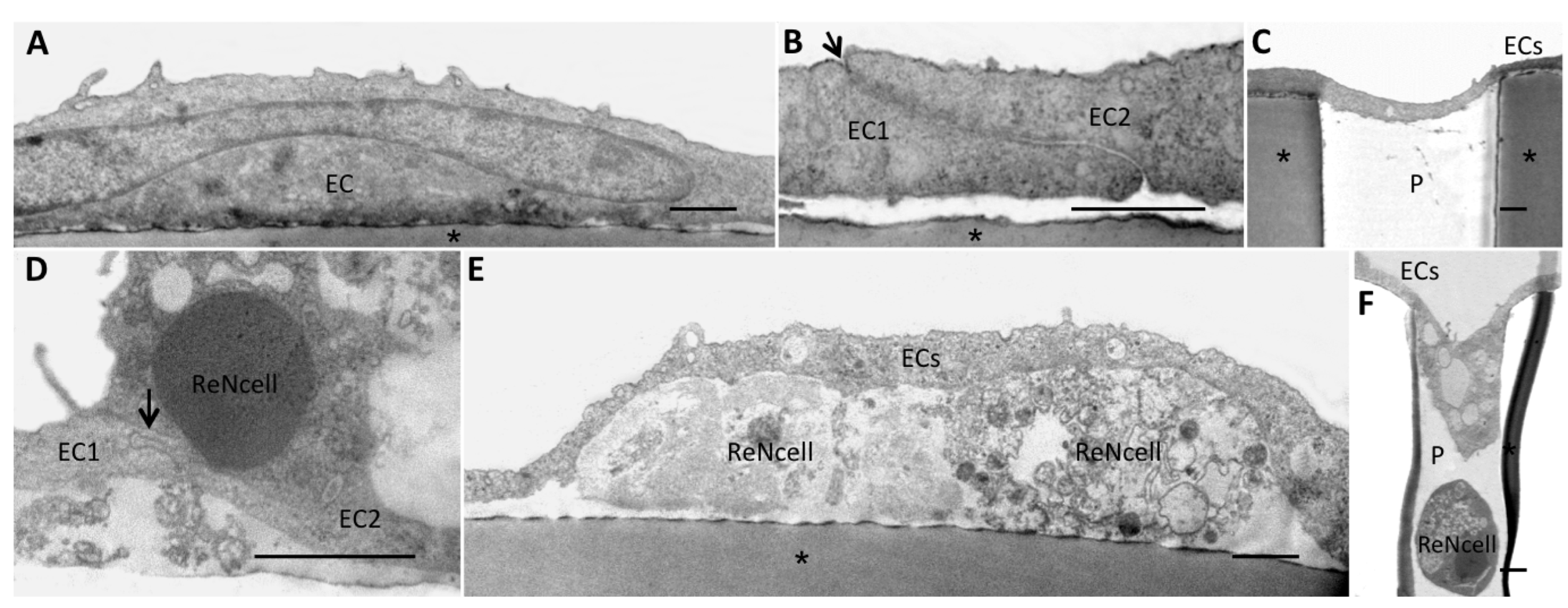

Supplement: Figure S2 — Transmission electron microscopy of a monolayer of RBMECs with transmigrating NSCs. A) Monolayer of RBMECs cultured on a Millicell filter with 8 µm pores. Left panel, observe the typical elongated morphology of an endothelial cell. Middle panel, border region between two neighboring endothelial cells; observe how the cells partially overlap. Right panel, view an elongated endothelial cell on top of a pore of the Millicell filter. B) ReNcells CX crossing a monolayer of RBMECs. Upper left panel, observe a ReNcell CX on top of two neighboring RBMECs. Lower left panel; observe two ReNcells CX located bellow the monolayer of RBMECs. Right panel, a ReNcell CX is found crossing through a Millicell pore. *, Millicell filter; arrow, paracellular pathway. EC1, endothelial cell one; EC2, endothelial cell two; P, Millicell pore. Bar = 1 µm. (TIF) [file pone.0060655.s002.tif]

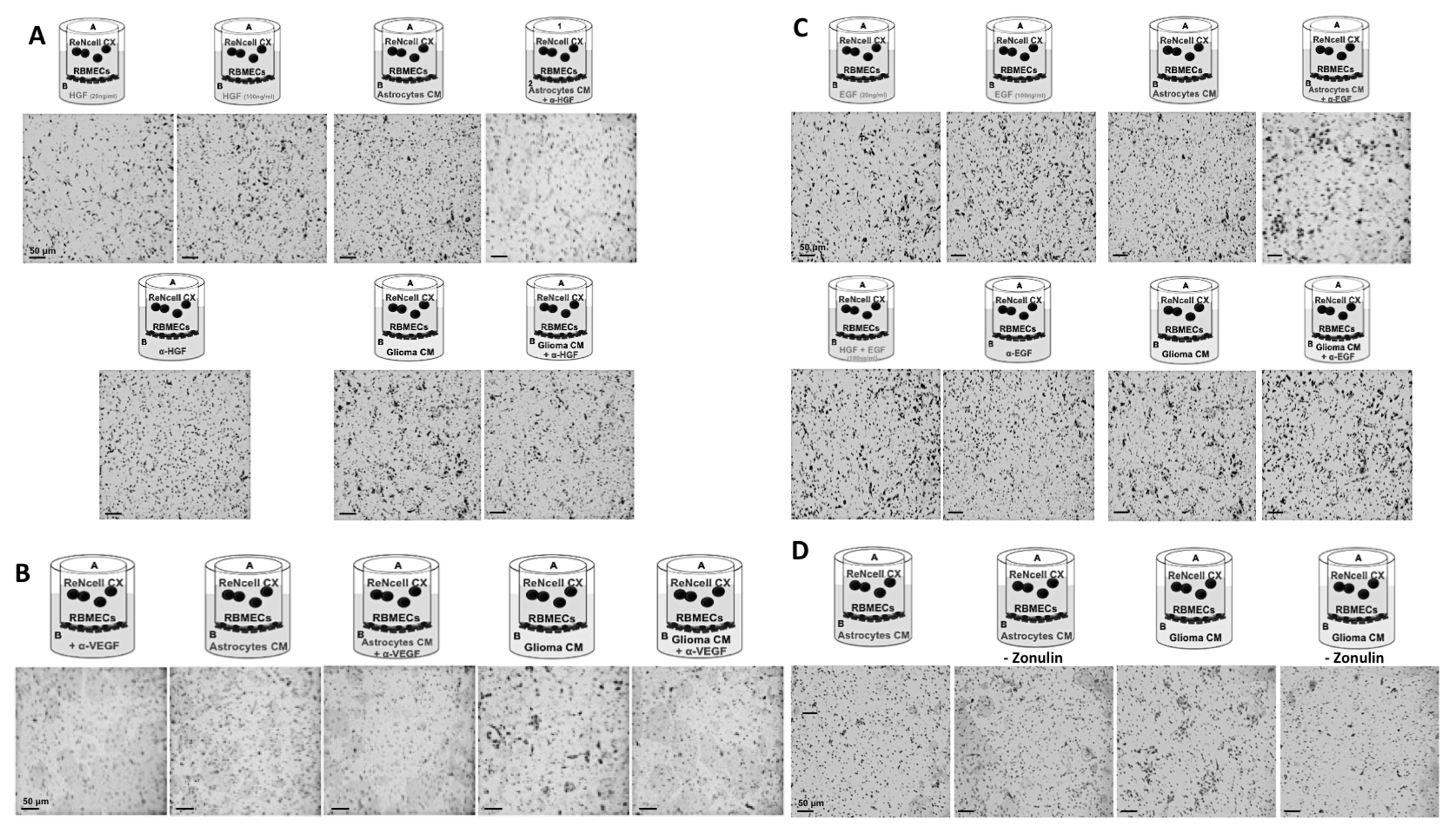

Supplement: Figure S3 — HGF, VEGF, zonulin, and the lack of EGF induce NSCs transmigration across RBMECs cultures. Representative light microscopy image of toluidine blue stained cells present on the basal surface of the filter and corresponding schemes illustrating each assay after the addition to the basal compartment of HGF or its neutralizing antibody (A), a neutralizing antibody against VEGF (B); EGF or its neutralizing antibody (C), and CM without zonulin due to specific IP (D) (TIF) [file pone.0060655.s003.tif]

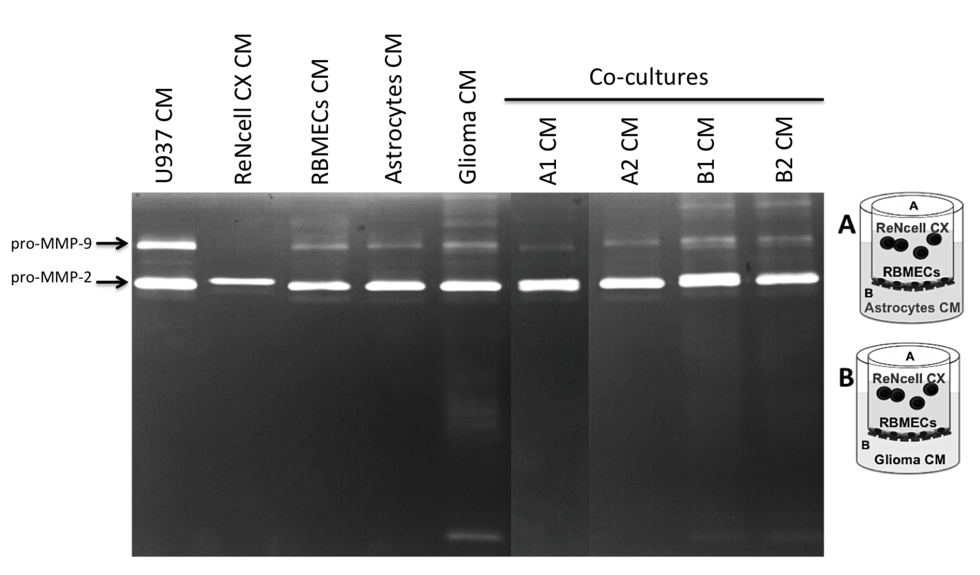

Supplement: Figure S4 — CM from glioma C6 and astrocytes have similar amounts of pro-MMP-2 and -9. CM derived from different cell cultures (ReNcells CX, RBMECS, astrocytes and glioma C6 cells) and from the co-cultures shown in the right hand side were electrophoresed in non-denaturing conditions in 8% polyacrylamide gels containing 1% gelatin. Proteolytic activity was induced by incubation in a Ca2+ containing buffer and proteolytic bands were visualized by Coomassie blue staining. CM from melanoma U937 cells was employed as standard of MMP-2 and -9 activities. A1CM, CM of assay A present in compartment 1; A2CM, CM of assay A present in compartment 2; B1CM, CM of assay B present in compartment 1; B2CM, CM of assay B present in compartment 2. (TIF) [file pone.0060655.s004.tif]

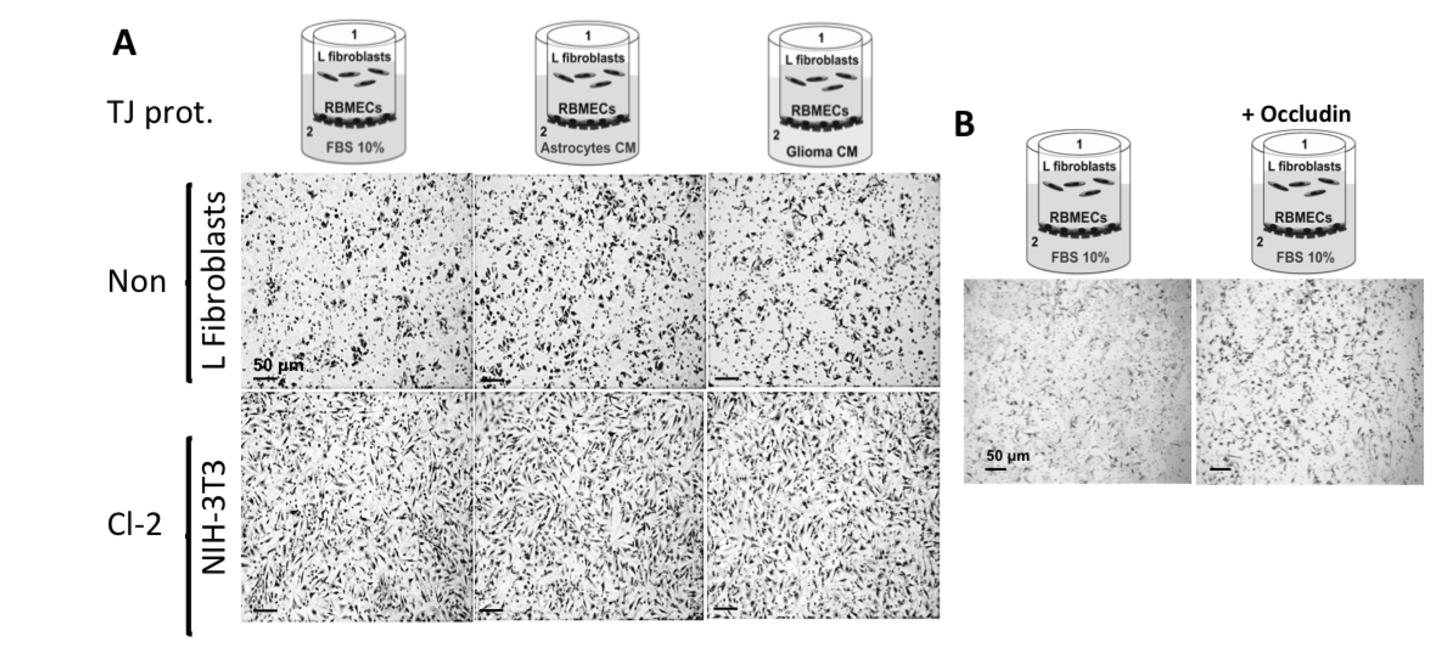

Supplement: Figure S5 — The expression of TJ proteins in fibroblasts enhances their transmigration across RBMECs. Transmigration assay with toluidine blue stained cells A) NIH-3T3 fibroblasts that express claudin-2, transmigrate across RBMECs in significantly higher amounts than L-fibroblasts, which do not express TJ proteins, independently of the CM present in the basal compartment. B) L-fibroblast that express occludin, transmigrate across RBMECs in significantly higher amounts than L-fibroblasts. (TIF) [file pone.0060655.s005.tif]

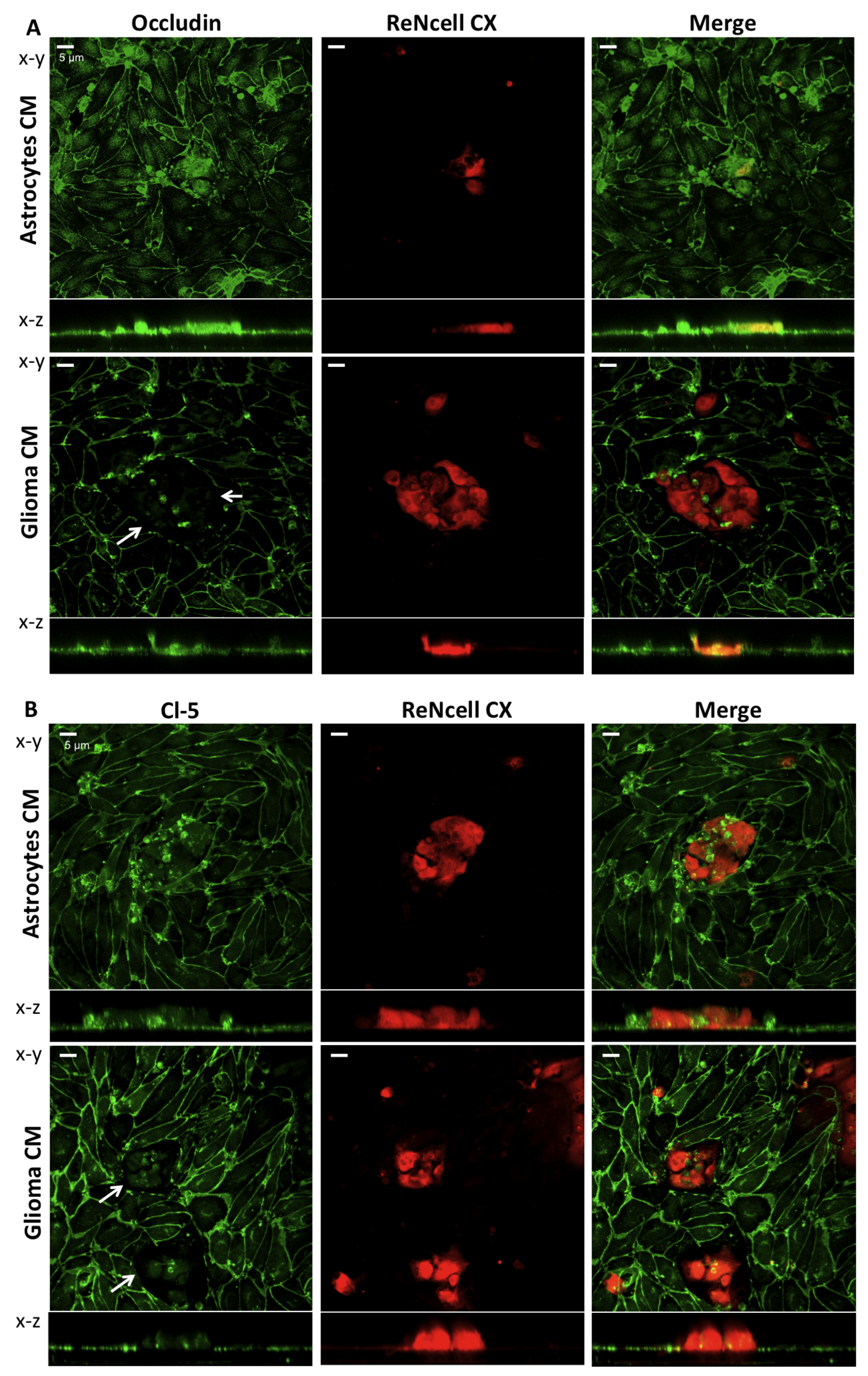

Supplement: Figure S6 — In RBMEC monolayers incubated with glioma C6 CM in the basal compartment, the expression of occludin and claudin-5 diminishes around the transmigrating ReNcells. Occludin (A) and claudin-5 (B) were detected with a specific antibodies followed by secondary antibodies coupled to FITC. Transmigrating ReNcells CX were stained in red with the cell tracker CMTMR. Arrows point to areas where occludin and claudin-5 expression is lost. (TIF) [file pone.0060655.s006.tif]

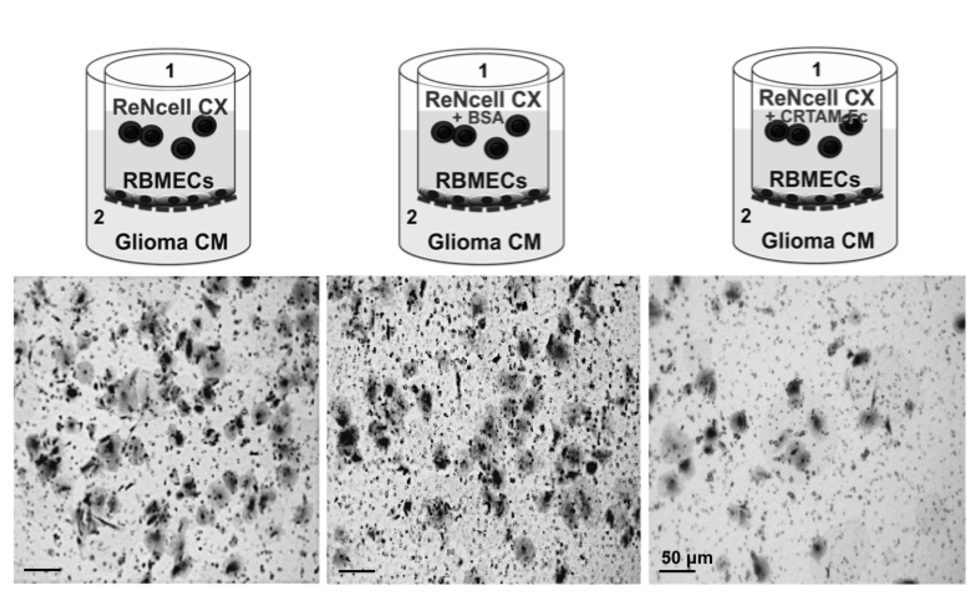

Supplement: Figure S7 — CRTAM mediated adhesion is important for the transmigration of NSC. CRTAM mediated cell-cell adhesion was competed by adding soluble human CRTAM (CRTAM-Fc) to the upper compartment of a Millicell insert with ReNcells CX. Upper left panel, scheme illustrating each assay; lower left panel, representative light microscopy image of toluidine blue stained cells present on the basal surface of the filter. (TIF) [file pone.0060655.s007.tif]

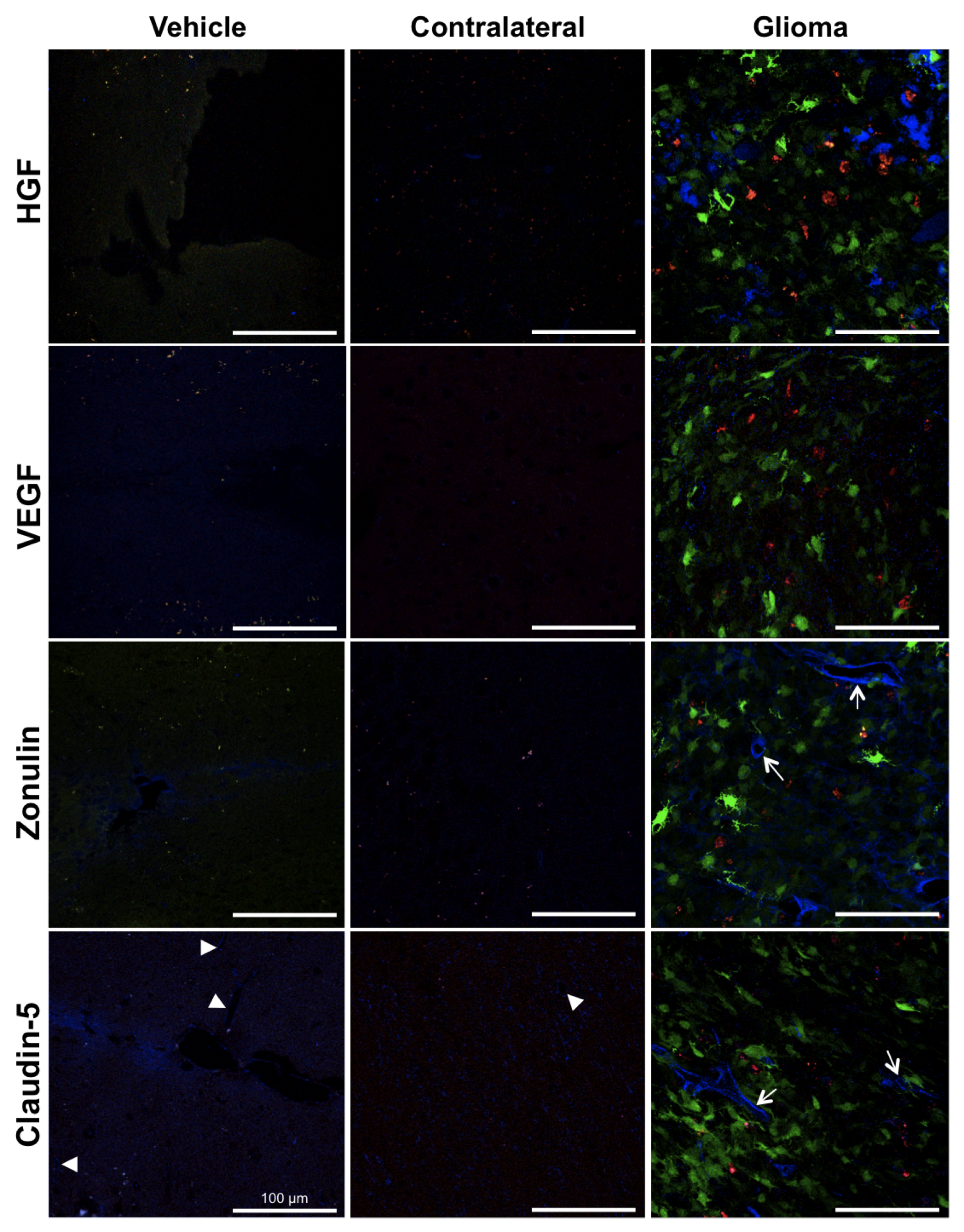

Supplement: Figure S8 — ReNcells CX injected into systemic circulation pass the BBB and reach intracerebral gliomas. Immunofluorescence detection (blue) of HGF, VEGF, zonulin/prehaptoglobin-2 and claudin-5, in brain slices of the stratum region of nude mice. The animals had previously been injected into the striatum with vehicle or glioma C6 cells containing the GFP sequence (green) and a week later received ReNcell CX stained with CMTMR (red) by injection into the tail vein. Brain slices were done one week later. Only at the tumor area, a strong signal of HGF and slight staining of VEGF is observed, while zonulin and claudin-5 strongly mark the cell borders of surrounding vessels (arrows). Zonulin is also present, albeit with very low intensity, in the area of the lesion in brains that had only received the vehicle (arrowhead). Claudin-5 gives a spotted pattern in the contralateral section (arrowhead) and stains, albeit with low intensity, the vessels in the vehicle only section (arrowhead). (TIF) [file pone.0060655.s008.tif]

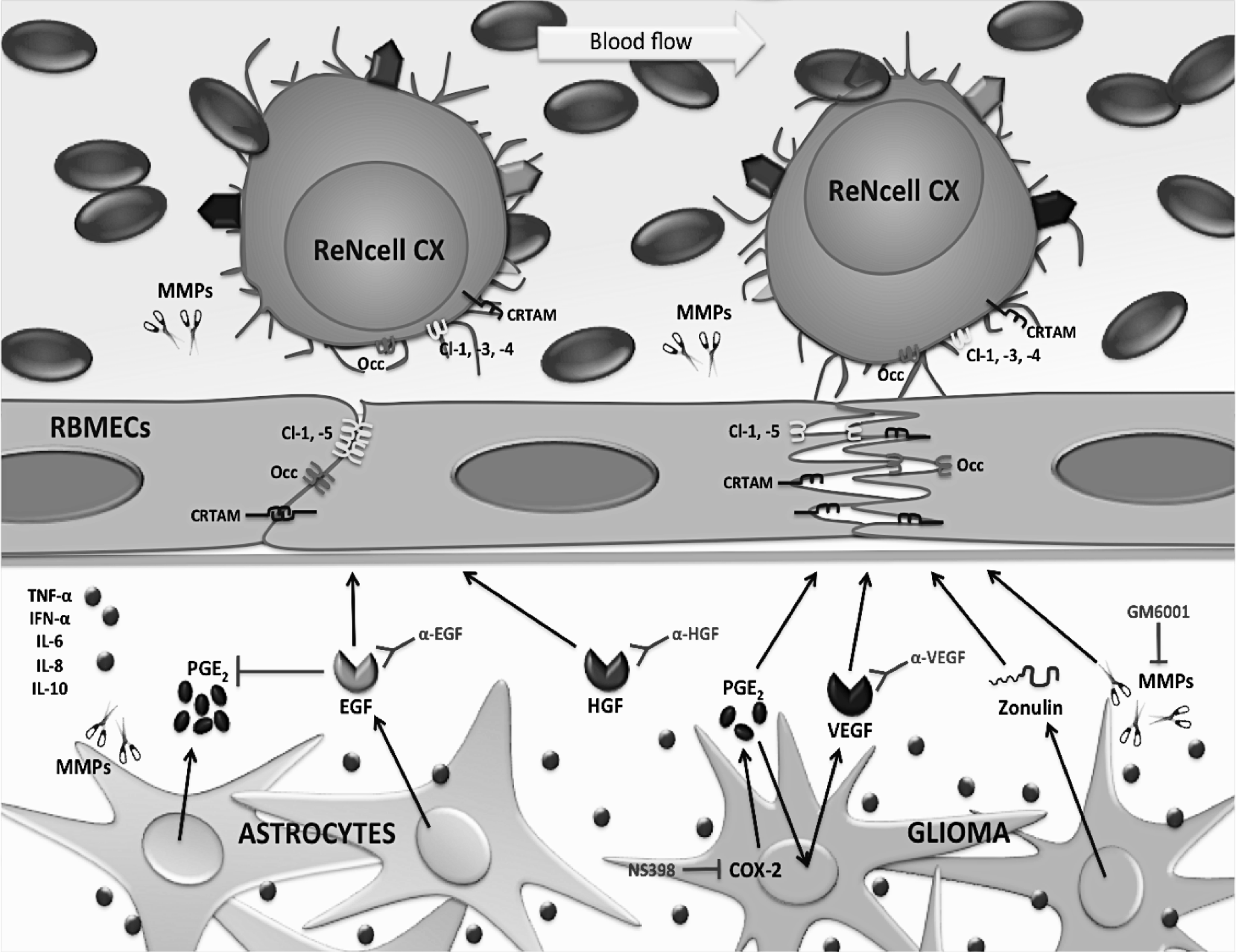

Supplement: Figure S9 — Schematic representation of the factors involved in the transmigration of NSC across the BBB. HGF, VEGF and zonulin secreted by glioma C6 cells, together with the absence of EGF, induce the transmigration of ReNcells CX across RBMECs. VEGF, zonulin, PGE2, and MMP different from -2 and -9, secreted by glioma C6 cells, open the BBB, whereas EGF secreted by astrocytes enhances TJ sealing. ReNcells CX express CRTAM, occludin and claudins 1, 3 and 4 that might facilitate their paracellular migration across RBMECs that have TJs formed by CRTAM, occludin and claudins 1 and 5. (TIF) [file pone.0060655.s009.tif]
